# Collisional Cross-Section Prediction for Multiconformational Peptide Ions with IM2Deep

**DOI:** 10.1021/acs.analchem.5c01142

**Published:** 2025-07-08

**Authors:** Robbe Devreese, Alireza Nameni, Arthur Declercq, Emmy Terryn, Ralf Gabriels, Francis Impens, Kris Gevaert, Lennart Martens, Robbin Bouwmeester

**Affiliations:** † VIB Center for Medical Biotechnology, 360916 VIB, Ghent 9052, Belgium; ‡ Department of Biomolecular Medicine, Faculty of Medicine and Health Sciences, Ghent University, Ghent 9052, Belgium; § BioOrganic Mass Spectrometry Laboratory (LSMBO), IPHC UMR 7178, University of Strasbourg, CNRS, Strasbourg 67000, France; ∥ Infrastructure Nationale de Protéomique ProFI—FR2048, Strasbourg 67087, France

## Abstract

Peptide collisional cross-section (CCS) prediction is complicated by the tendency of peptide ions to exhibit multiple conformations in the gas phase. This adds further complexity to downstream analysis of proteomics data, for example for identification or quantification through feature finding. Here, we present an improved version of IM2Deep that is trained on a carefully curated data set to predict CCS values of multiconformational peptides. The training data is derived from a large and comprehensive set of publicly available data sets. This comprehensive training data set together with a tailored architecture allows for the accurate CCS prediction of multiple peptide conformational states. Furthermore, the enhanced IM2Deep model also retains high precision for peptides with a single observed conformation. IM2Deep is publicly available under a permissive open-source license at https://github.com/compomics/IM2Deep.

## Graphical abstract

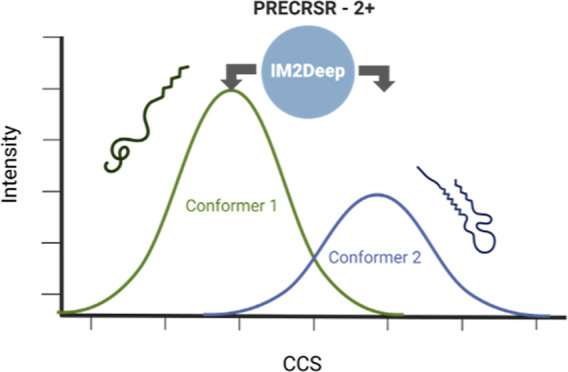

## Introduction

Recent advances in LC–IM–MS/MS technology have strongly enhanced identification capabilities in complex proteomics workflows.^1–3^ Traditionally, LC–MS/MS systems solely depended on liquid chromatography and mass analyzers for peptide separation and selection before fragmentation. In contrast, MS instruments incorporating ion mobility (IM), like the timsTOF series, enable gas-phase ion separation with ion mobility spectrometry (IMS) and use parallel accumulation–serial fragmentation (PASEF) technology to increase sensitivity and acquisition speed.^4^

The inversed reduced IM and the mass-to-charge ratio can be used to calculate the collisional cross section (CCS) of a peptide ion using the Mason-Schamp equation.^5^ CCS represents the rotationally averaged effective area with which an ion collides with a neutral gas and is closely tied to the ion’s chemical structure and three-dimensional conformation. This characteristic is useful for improved identification confidence upon comparing predicted and observed CCS values.^6,7^ Predicted CCS values can also be used for more accurate quantification through feature finding^8^ and to prioritize acquisition time.^9^ The potential benefits of predicted CCS values led to the development of various machine learning models based on amino acid- or atom-level features and physicochemical properties.^6,7,10^ However, current models overlook the possibility of peptide ions adopting multiple conformations in the gas phase (Figure 1), as these models are often trained on the most abundant conformer within a data set. This can result in the exclusion of valuable data and biases a model toward the most prevalent conformers in each experiment, inherently assuming that the dominant conformer in one experiment will consistently dominate in others, which is not a foregone conclusion and might result in inaccurate prediction.

**Figure 1:**
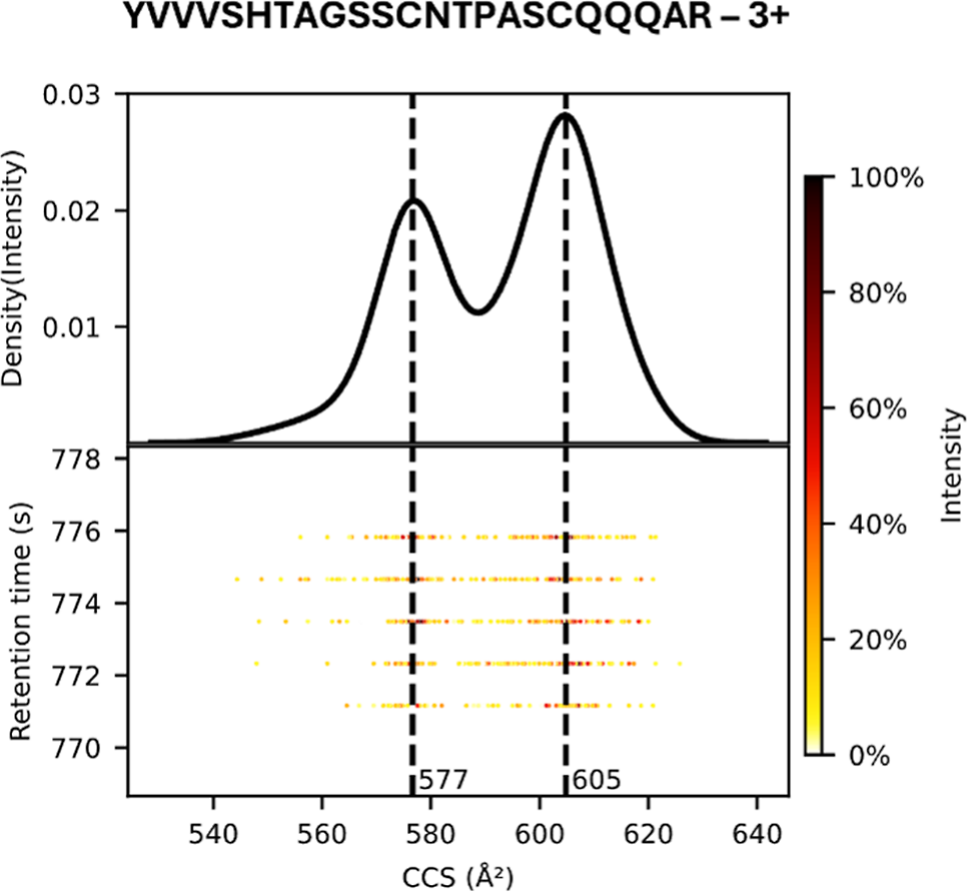
IM distribution of a representative peptide ion (UniProt ID of inferred protein: O75594, data from PRIDE accession PXD046507). The upper panel shows the IM distribution with multiple distinct peaks, indicating the presence of multiple conformations. The lower panel displays the ion intensity plotted against retention time (s) and CCS (Å^2^). MaxQuant reported two CCS features for this precursor, one at 577 Å^2^ and one at 605 Å^2^.

Previous studies have identified several factors contributing to the multiconformationality of peptide ions in LC–IM–MS/MS experiments, such as the solvent composition,^11^ activation voltage,^12^ cis–trans isomerization of proline residues,^13^ and charge localization within the precursor.^14^ These factors can result in multiple conformers that are distinguishable by IMS–MS.

The increasing availability of TIMS data sets facilitates the development of deep learning models that can effectively predict CCS for multiconformational peptides, as multiple data sets can be efficiently aggregated into a large quantity of high quality training examples, even if multiconformational peptide ions in a single data set are rare.

We recently introduced IM2Deep, a deep learning-based CCS predictor that builds on the principles of DeepLC.^7,15^ IM2Deep encodes peptidoforms at the atomic composition level, enabling precise CCS predictions for peptides with modifications not encountered during training. In this study, we present an improved version of IM2Deep designed to predict CCS values for peptide ions exhibiting multiple conformations in the gas phase. This was achieved by modifying its architecture to support multi-output predictions and using transfer learning to tailor the model for a smaller data set of multiconformational peptide ions. These changes enable IM2Deep to accurately predict CCS values for multiconformational peptides.

## Methods

### Data Sets

We searched the PRIDE public proteomics data repository for data sets generated using timsTOF Pro and timsTOF Pro 2 instruments, retaining those with readily available identification output processed using MaxQuant, as this includes CCS annotations.^16^ To ensure a large sequence variety in our data set, we included data originating from a large number of distinct species (*n* = 16). In total, we collected MaxQuant evidence output from 30 different PRIDE projects,^10,17–43^ totaling 1248 LC–IM–MS/MS runs for further processing. Additionally, we included immunopeptidomics data (366 LC–IM–MS/MS runs) from Gravel et al.,^44^ to enhance the model’s performance on nontryptic peptides, and on singly charged peptides. This data set was searched as described in Declercq et al.^7^ An overview of data sets can be found in Supporting Information Table S1.

### Creation of a Multiconformer Peptide Ion Data Set

This section details the steps taken to aggregate the separate experimental data sets into a unified data set of multiconformational peptides (Supporting Information Figure S1). First, only peptide ions identified by an MS/MS spectrum and with distinct MS1 features were kept for further processing. Then CCS values across all LC-IM-MS/MS runs are aligned in each experiment by calculating a charge-specific linear offset (*y* = *x* + *b*) between overlapping peptide-charge state pairs. This alignment method is similar to the one described by Meier et al.^10^ For each peptide-charge state pair, the CCS of the identification with the highest intensity was used as the alignment reference, but CCS values of the less abundant identifications were also aligned and retained. Beginning with the run containing the highest number of identifications, all other runs were sequentially aligned and added to the data set in descending order of identifications. To ensure good alignment, runs with less than 100 overlapping peptide-charge state pairs with the aligned data set (*n* = 284) were ignored.

Following CCS alignment, conformers were identified within each LC–IM–MS/MS run. Identical peptide ion identifications exhibiting a larger than 2% difference in CCS within the same run were considered distinct conformational states, as suggested by previous research.^10^

To increase the confidence in accurately identifying conformers, we filtered the data set by checking for recurring conformers across multiple separate runs. Specifically, we compared the CCS values reported for each peptide ion in each run. Only multiconformational peptide ions with multiple CCS values that could be matched to corresponding values in other runs, within a 2% tolerance, were retained. The mean CCS value for each conformer was retained in the data set. Peptide ions with charge state 5+ and 6+ were excluded from further analysis, because of their low occurrence (*n* = 5, combined).

Peptide ions that did not meet the above criteria were classified as uniconformational and placed in a separate data set. This data set underwent additional filtering to confirm that multiconformational peptide ions, where distinct conformers appeared only between runs (and not within the same run), were excluded. Thus, only peptide ions with CCS values showing no deviation greater than 2% between aligned runs were included in the uniconformer peptide ion data set.

### IM2Deep Architecture for Multiconformer CCS Prediction

The architecture of the original IM2Deep model is described in Declercq et al.^7^ To enable multiconformer CCS prediction, this architecture was adapted to support multi-output prediction. Instead of a single-output node, IM2Deep now features a branched output with two final dense layers, each ending in a single-output node, producing a distinct CCS prediction. We trained the multi-output IM2Deep models both with and without a pretrained single-output model as foundation. The single-output model had been trained on the data set used in Declercq et al.^7^ Pretrained model weights were loaded into the shared layers of the multi-output model prior to training, with all parameters remaining fully trainable.

The multi-output IM2Deep models were trained and evaluated on the multiconformer data set, which was randomly split into 81% training, 9% validation, and 10% testing sets. Training continued for a maximum of 500 epochs, with early stopping based on validation set performance to prevent overfitting. The custom loss function used to train the multi-output model, which ensures that each prediction corresponds closely to a distinct target, is outlined in the Supporting Information Methods. Supporting Information Table S2 shows the hyperparameters of the final multi-output IM2Deep model.

## Results

### Description of the (Multi)­Conformer Data Set

In this study, we compiled identification data from 1614 LC–IM–MS/MS runs across 31 PRIDE projects to determine peptide ion conformers. To enhance the reliability of our data set, we excluded conformers not identified in multiple runs (Supporting Information Figure S3).

After filtering the data set, the final multiconformer data set included 33,468 unique peptidoform-charge state pairs. This is 3.1% of the size of the uniconformer data set (*n* = 1,089,294), which is in line with previously published estimates.^6,10^ The number of observed conformational states for a single peptide ion in the multiconformer data set reached as high as six (*n* = 4, Supporting Information Figure S4).

We identified differences in the physicochemical properties of uni- and multiconformational subsets of peptide ions (Figure 2). Notably, multiconformational peptide ions tended to be longer (Figure 2A) and carried higher charges (Figure 2B). These observations support findings by Meier et al., who reported that prediction models trained to estimate only one CCS value show a bias, particularly affecting the accuracy for longer, 3+ and 4+ charged peptides.^10^

**Figure 2:**
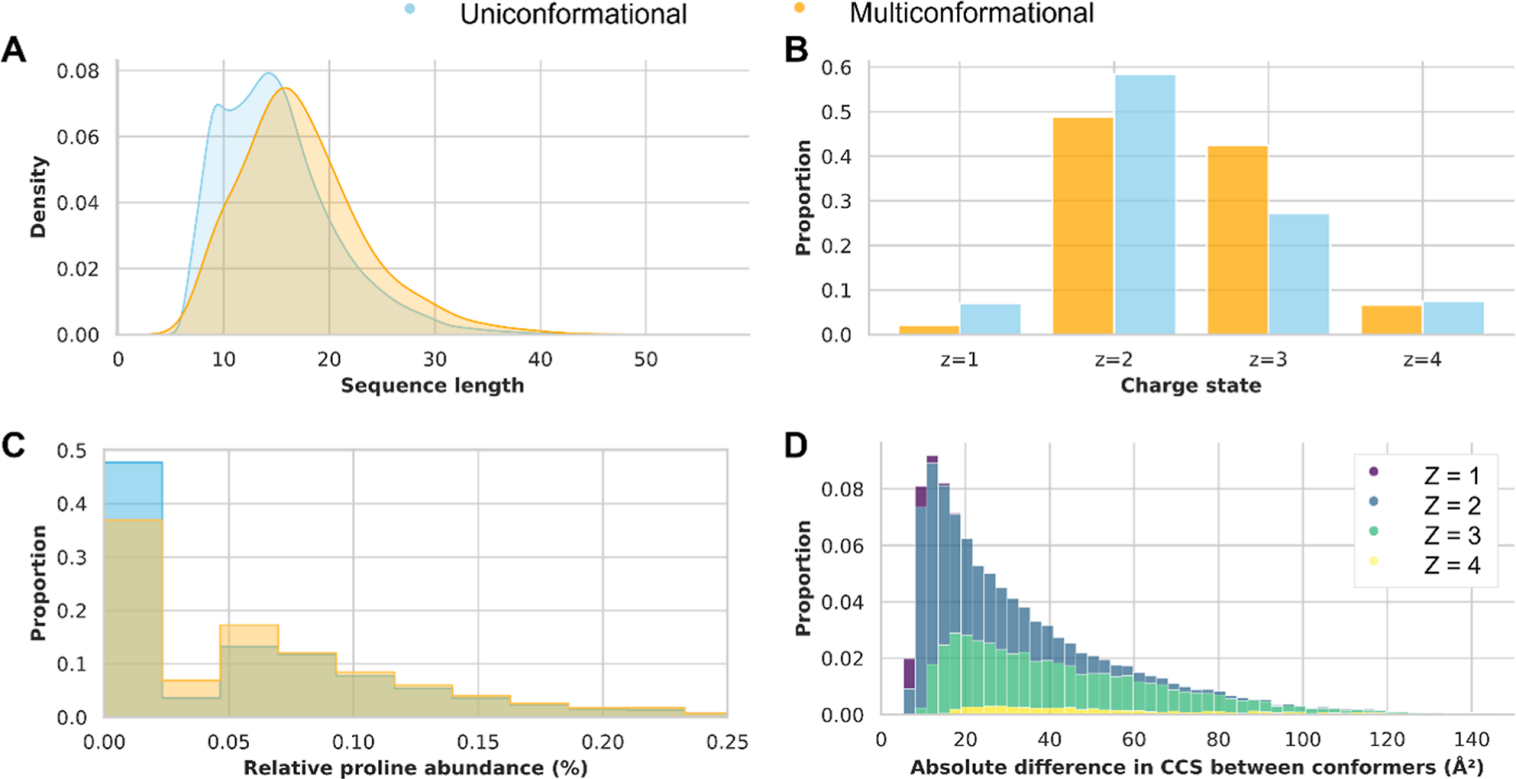
Physicochemical property differences between uniconformational (blue) and multiconformational (orange) peptides. (A) Sequence length distribution (B) distribution of charge states (C) relative abundance of proline residues. (D) Stacked histogram showing the distribution of absolute differences in CCS between conformers for peptide ions with different charge states.

Given the hypothesis that cis–trans isomerization of proline residues could contribute to multiconformationality, we examined the presence of proline within the two subsets. Indeed, we identified an enrichment in proline presence within the multiconformational peptides (63.1%), compared to peptides not exhibiting multiple conformations (52.3%). Additionally, the relative frequency of proline residues -defined as the number of prolines divided by the length of the sequence-is also higher in the multiconformer subset (Figure 2C). Furthermore, peptides with higher charge states exhibited greater differences in CCS between conformers (Figure 2D, Supporting Information Figure S5), with a broader distribution of CCS differences observed among highly charged peptide ions. Small amino acids, such as alanine, glycine and valine also occur more often in the multiconformational peptides, while longer amino acids such as glutamic acid, lysine and arginine had a lower relative presence (Supporting Information Figure S6).

### Multi-output IM2Deep for Two Conformer CCS Prediction

Using the multiconformer data set, filtered for peptide ions exhibiting two conformations (*n* = 30,344), we trained a new version of the IM2Deep model specifically adapted for multi-output prediction, targeting two CCS values corresponding to each conformer. We employed two training strategies: one involved training a model from scratch, while the other utilized a fine-tuning approach, where weights from a pretrained single-output IM2Deep model were loaded into the shared layers of the multi-output model before training started. This method leverages the features learned from the pretrained model to enhance prediction accuracy, a strategy that has previously demonstrated success in challenging peptide property predictions.^45,46^

Learning curves on the validation set reveal that the fine-tuned model outperforms the newly trained model (Supporting InformationSupplementary Figure S7). The test set results further corroborate these findings (Figure 3), with the fine-tuned model showing superior prediction accuracy compared to the model trained with random initialization. Importantly, the median relative error for both CCS predictions in the fine-tuned model remains well below the 2% threshold used to define multiconformationality. Approximately 94% of predictions for the small conformer and 97% for the large conformer have an error smaller than the median difference between the observed CCS values of the two conformers (Supporting Information Figure S8). This suggests that the model can effectively distinguish between different conformers and accurately predicts their corresponding CCS values with high precision. For charge state 4 peptide ions, the predictive accuracy can be more variable, which likely stems from the fact that the number of training peptide ions carrying this charge state is low. While both training and testing data sets contained predominantly tryptic peptides, the predictions for nontryptic (immuno)­peptides are even more accurate (Supporting Information Figure S9).

**Figure 3:**
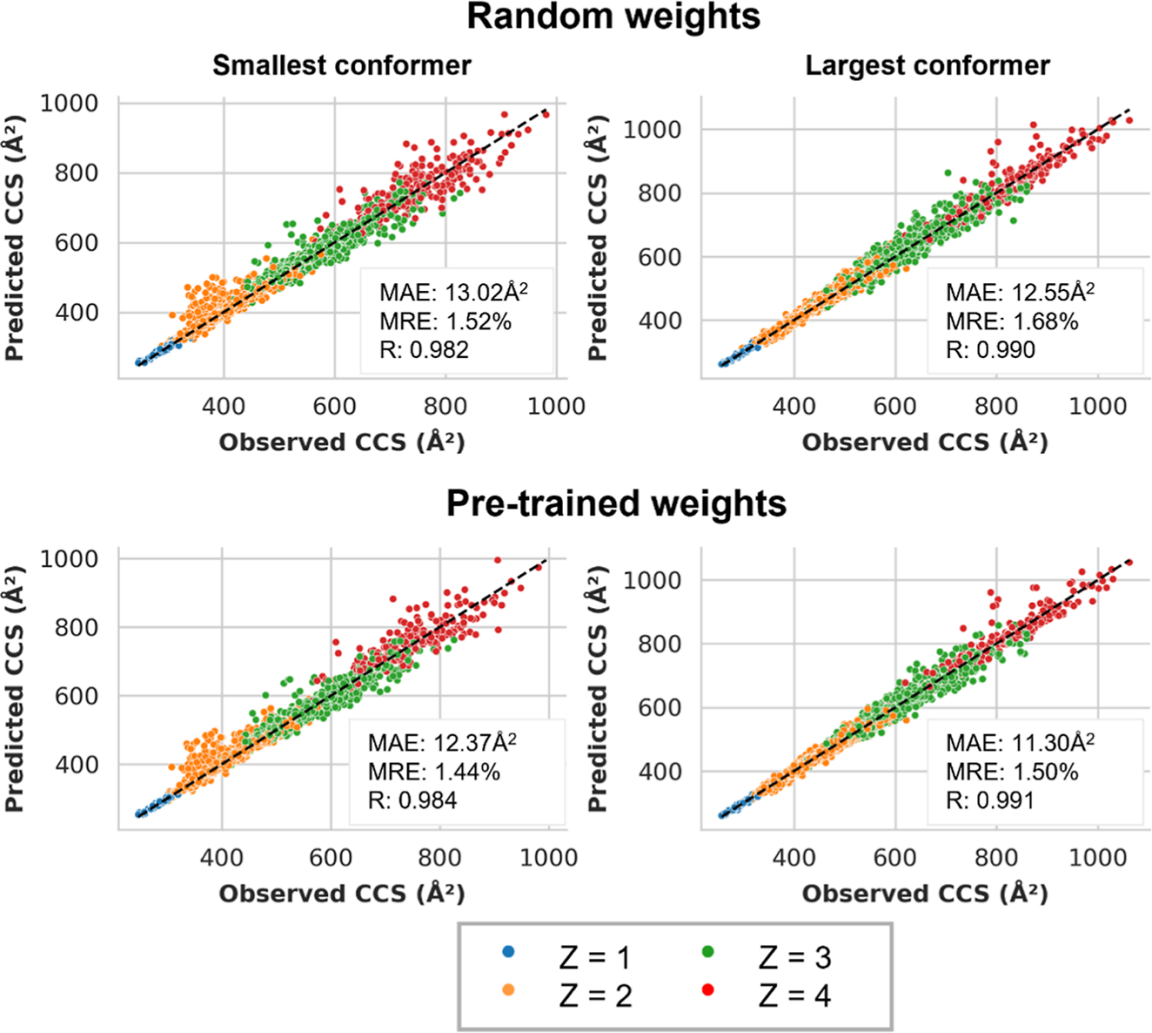
Scatter plots comparing predicted versus observed CCS values for the multi-output IM2Deep model. The top row shows the performance of the model trained with random weight initialization, while the bottom row displays the performance of the model fine-tuned with pretrained weights from a single-output IM2Deep model. Each point represents a peptide ion, with color indicating its charge state. The diagonal line represents the ideal scenario where predicted CCS values match observed values. The left plots correspond to the first, smaller CCS prediction, and the right plots to the second, larger CCS prediction of each multiconformational peptide ion.

### Evaluating Model Robustness and Comparison with Baseline Models

To ensure that an additional second prediction does not artificially improve its performance, a comparison is made with two baseline models: (1) a baseline model trained on CCS values from peptides exhibiting only one conformation (the uniconformer data set, see methods); (2) a baseline model trained on the CCS values of one randomly selected conformation from each peptide in our multiconformational training set. Despite being trained on only one target CCS value per peptide, the architecture of these baseline models have two output nodes and thus allow for two predictions. These baseline models serve as benchmarks to evaluate the benefits of training a model on multiple CCS values for multiconformational predictions. The loss function used to train the baseline models is calculated as the sum of the mean absolute errors between each prediction and the target CCS.

As illustrated in Figure 4A, the finetuned model strongly outperforms both baseline models in predicting multiple CCS values, with marked improvements in both MAE and Pearson correlation coefficients. These results underscore the model’s ability to effectively capture the multiconformational nature of these peptides, owing to its training on multiple conformations, and not just because of the capacity of the model to make two predictions per peptide ion.

**Figure 4:**
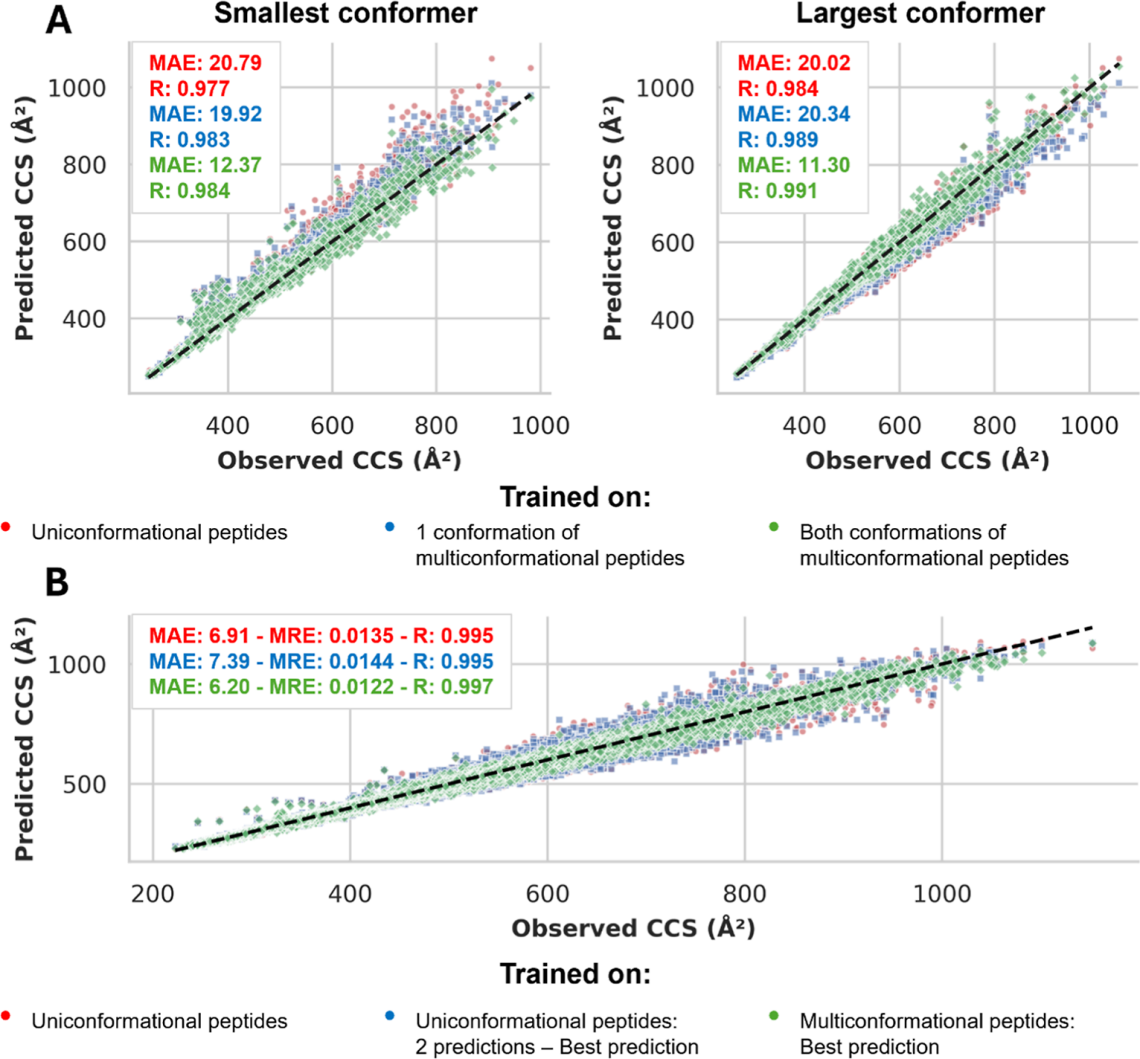
(A) Scatter plots comparing predicted versus observed CCS values for the multi-output IM2Deep model and two baseline models, each trained on different data sets. Each dot corresponds to a peptide ion, with the dashed line representing the ideal scenario where predicted CCS values match observed values. (B) Scatter plot comparing predicted versus observed CCS values for uniconformational peptides using the different models.

An ideal multiconformational model should also accurately predict CCS values for peptides where only one conformation is observed. To assess this, we compared the accuracy of the closest prediction made by the finetuned multiconformational model to that of a model trained exclusively on uniconformational peptides, on a test set of uniconformational peptides. Although the multiconformational model has the advantage of making two predictions and selecting the best one, we controlled for this potential bias by allowing the baseline model trained on uniconformational peptides to also make two predictions, again selecting the best one.

The results, shown in Figure 4B, indicate that the multiconformational model, despite being trained on peptides with multiple conformations, maintains or even improves its accuracy in predicting CCS values for peptides with only one observed conformation. This improvement likely stems from the model’s ability to capture important structural information that is not fully represented in models trained on a single CCS value.

We also compared the IM2Deep model trained on either uni- or multiconformational peptide ions with the model presented in Meier et al. to predict CCS values for an independent data set with peptides originating from a species not included in training data of either model (, PXD056927 ^47^). While the IM2Deep model trained on uniconformational peptides showed comparable performance, the model trained on multiconformational peptides showed a notable improvement (Supporting Information Figure S10).

### Evaluating the Advantages of the Multiconformer Model on an Independent Data Set

To quantitatively assess the extent of multimodal IM distributions in current LC–IM–MS/MS data sets and demonstrate the capabilities of the multi-output IM2Deep model, we generated extracted ion mobilograms (XIMs) for identified peptide ions from two independent data sets.^48,49^ To evaluate multiconformationality, we applied a peak-picking algorithm to identify peaks in the IM dimension (Supporting Information Methods). As shown in Supporting Information Figure S11, a considerable proportion of XIMs display bi- or multimodal distributions, with this pattern being highly reproducible across technical replicates. When considering peaks with an intensity greater than 10% of the highest peak, approximately 35% of the XIMs exhibit multimodal behavior. Tightening this intensity threshold to 75% still reveals that around 8% of XIMs are multimodal. These percentages are higher than what was observed during the data set collection for the model (∼3%), which can in part be explained by the relatively stringent criteria for inclusion into the model data set. Furthermore, coeluting isobaric peptides could cause overlapping IM distributions in some instances.^50^ Finally, when secondary peaks are considered at low thresholds, this difference might also be caused by noise peaks that do not reflect actual conformers. However, since there is still a difference when secondary peaks are considered at high thresholds, the capability of search engines to identify peptide ions with multiple IM features warrants further investigation. For further analysis, we retained secondary peaks with intensities exceeding 25% of the main peak’s intensity. As an example, we show a peptide ion displaying two distinct peaks in the IM distribution indicating the presence of two conformations (Figure 5A). For this peptide ion, the original IM2Deep model successfully predicts the CCS for one of these conformers (red), while MaxQuant reports the CCS of the other, more intense conformer (black). In PSM rescoring, where the observed CCS values are compared to predicted ones, this discrepancy could lead to the penalization and potential exclusion of the PSM, even though IM2Deep made an accurate prediction. In contrast, the multiconformational model accurately predicts the CCS values for both conformations, eliminating this issue when the closest prediction to the observed CCS is used for rescoring.

**Figure 5:**
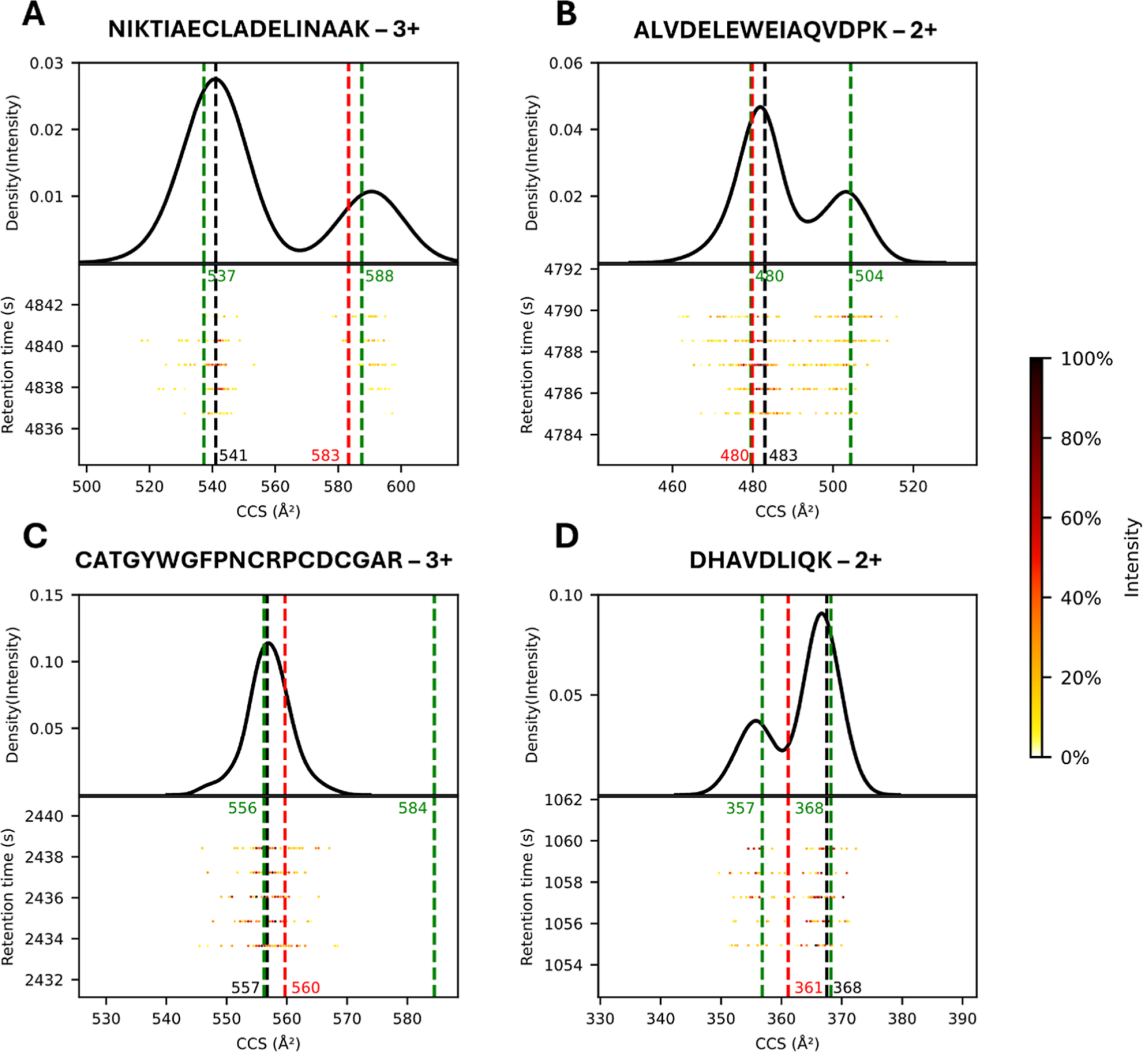
Ion mobility distributions of selected peptide ions from PXD046507,^48^ highlighting the capabilities of the multi-output IM2Deep model. Dashed lines represent CCS value reported by MaxQuant (black), classical single-output IM2Deep prediction (red) and CCS predictions made by multiconformational model (green). (A) Peptide ion (UniProt ID of inferred protein: P46782) showing two distinct peaks in the ion mobility distribution, where the classical model predicts the CCS of the nonreported conformer. (B) Peptide ion (UniProt ID: Q9Y2B0) where the multiconformational model accurately predicts CCS of a conformer not identified by MaxQuant. C) Peptide ion (UniProt ID: O15230) showing a single dominant conformation, accurately predicted by the multiconformational model. (D) Peptide ion (UniProt IDs: P0C0L4 and P0C0L5) where classical IM2Deep model prediction appears to be the average of two conformations. Observed and predicted CCS values are rounded to the nearest integer for simplicity.

An interesting advantage of the new multiconformational model is its ability to accurately predict CCS values for conformations not identified by the search engine. For example, as shown in Figure 5B, while MaxQuant reported the CCS value of the most intense conformer, the multiconformational model accurately predicted the CCS value of a less intense, unreported conformer. This capability offers deeper insights into the structural dynamics and conformational diversity of peptide ions in the gas phase.

In most cases, peptide ions exhibit a single conformation (Figure 5C). As previously demonstrated, the multiconformational model still makes accurate predictions for these peptide ions. When the best prediction is selected, the second prediction might be redundant, but could also represent an accurate prediction of an unobserved conformer.

As discussed by others, a maximum likelihood estimation approach to CCS prediction typically converges to the mean CCS value of all conformers when no prior conformer filtering is performed and a mean value for each peptide ion is retained in the final training set.^6^ Previous approaches have tried to avoid this by retaining only the most intense conformer in each data set. However, when data sets are combined, and CCS values are averaged over multiple data sets, this assumes that the most intense conformer of each peptide is consistent across all experiments, which is often not the case.^11,12^ To measure the extent of this problem, we compared XIMs of identical peptide ions across two technical replicates. For ∼ 35% of peptide ions with bimodal XIMs, the order of the intensity of the two peaks was found to reverse (Supporting Information Figures S12 and S13). This percentage drops to around 20% when the secondary peaks in both replicates are required to be less than 90% as intense as the main peak, indicating that some peptide ions have two peaks in the IM distribution with similar intensities. Notably, we observe that for about 20% of the peptide ions flagged as bimodal, the original single-output IM2Deep model predicts a CCS value closer to the mean of the two conformers than either of the individual CCS values (exemplified in Figure 5D). This observation likely stems from an erroneous averaging of CCS values from different conformers when different data sets are combined into a training set and only the most abundant conformer is retained from each data set. Although this observation was made across technical replicates, the problem of inconsistent peak intensities is likely to be even more prevalent when comparing XIMs of peptide ions across different experimental conditions. This variability in conformer intensity ordering across replicates may have implications for quantification accuracy, which should be investigated further. Our novel approach assumes consistent ordering of conformer intensities only for calculating linear offsets to align CCS values between data sets, where the impact of less common multiconformational peptides should be minor. During training, however, no hierarchical order is imposed on conformers; instead, they are matched across data sets based on their CCS values, with an average value retained for each conformer. This approach allows for more accurate and flexible predictions, accounting for the possibility of multiple conformations that may not be consistent across experiments.

It is important to note that none of the peptide ions used as examples in this analysis were included in the training data for the multiconformational model.

## Discussion

In this study, we developed a new multi-output IM2Deep model designed to predict CCS values for peptides that exhibit multiple conformations.

Here, we limited the training of the multi-output IM2Deep model to two CCS values due to the size limitations of our training set. While this approach provides important improvements, it is evident that predicting CCS values for more conformations would further enhance performance for peptides showing more than two conformations (Supporting Information Figure S13A). Our current model architecture is flexible, requiring only slight adjustments to the architecture and training procedure to accommodate these higher-order predictions.

Another intriguing aspect is the feasibility of predicting whether a peptide ion will exhibit multiconformational behavior. It might be the case that all peptide ions have the potential to exhibit multiple conformations depending on experimental conditions. Further investigations into the factors that influence multiconformationality are essential to facilitate the development of such predictors. One possible direction is to model multiconformational behavior as a classification task, using sequence-derived features, physicochemical properties, and experimental and instrument-specific metadata (e.g., solvent conditions) as input features. Combining such a predictor for multiconformational behavior with a multi-output CCS predictor can be a highly effective tool for improved identification confidence. In addition, while our current model does not attempt to rank the predicted conformers by their relative abundance -as this varies between LC–IM–MS/MS runs-future updates to IM2Deep could implement an additional fine-tuning step where features based on experimental conditions are used to score each predicted conformation.

However, large challenges remain, particularly in distinguishing between conformers with overlapping ion mobility signals and minimal CCS differences (Supporting Information Figure S13B). Because search engines typically report apex CCS values, future research should focus on accurately linking ion mobility measurements to identified peptide ions. This would enable the prediction of entire ion mobility distributions rather than discrete CCS values. Accurately capturing these distributions would require advanced modeling techniques capable of handling the inherent complexity and variability in ion mobility data. Continued data collection and sharing within the proteomics community are vital for the advancement of CCS prediction models by providing the necessary data diversity and volume.

## Conclusion

In this study, we enhanced IM2Deep by implementing a multi-output deep learning approach to predict collision cross sections for peptide ions exhibiting multiple conformations. Our findings demonstrate that the enhanced IM2Deep model accurately predicts CCS values for various conformers while maintaining high precision for peptides with a single observed conformation. Improved CCS prediction accuracy can lead to more confident identification of peptides. Furthermore, the ability to predict CCS for multiple conformations can offer deeper insights into the structural dynamics and behavior of peptides in the gas phase.

## Supplementary Material



## Data Availability

IM2Deep is open source under the permissive Apache-2.0 license and is freely available as a Python package through PyPI and Bioconda. The source code is available on GitHub at https://github.com/compomics/IM2Deep. IM2DeepTrainer, a Python package used to train new (multi-output) IM2Deep models is open source under the permissive Apache-2.0 license and is freely available as a Python package on PyPI. The source code is available on GitHub at https://github.com/rodvrees/IM2DeepTrainer. All data and scripts required to reproduce the presented results are available on Zenodo at 10.5281/zenodo.14886715.

## Notes

Additional methodology; (Table S1) overview of the public data sets used for further processing; (Figure S1) processing workflow for creation of multiconformational peptide ion data set; (Table S2) hyperparameters of final IM2Deep model; (Figure S2) Validation accuracy of models trained with varying difference weights; (Figure S3) distribution of conformer counts identified across multiple LC–IM–MS/MS runs; (Figure S4) distribution of conformers per peptidoform-charge state pair; (Figure S5) distribution of relative CCS differences between conformers; (Figure S6) comparison of amino acid abundances between multi- and uniconformational peptides; (Figure S7) performance comparisons between model trained from scratch and transfer-learned model; (Figure S8) distribution of absolute relative errors for predictions on the multiconformational peptide ion test set; (Figure S9) performance of the model on tryptic and nontryptic peptides; (Figure S10) comparison with model from Meier et al.;^10^ (Figure S11) distribution of XIMs with one, two or more than two detected peaks; (Figure S12) relationship between log-transformed ratio of IM peak intensities across two technical replicates; (Figure S13) XIMs from a multiconformational peptide ion across three replicates; (Figure S14) examples of remaining challenges in CCS prediction (PDF)
