# Supplementary material for: Collisional Cross-Section Prediction for Multiconformational Peptide Ions with IM2Deep
Source: Anal Chem. 2025 Jul 8;97(28):15113–21. doi: 10.1021/acs.analchem.5c01142 (PMC12291050; doi:10.1021/acs.analchem.5c01142)
Supplement: Supplementary file 1 [file ac5c01142_si_001.pdf]

Supporting information for "Collisional cross-section prediction for multiconformational peptide ions with IM2Deep"

Robbe Devreese[1,2], Alireza Nameni[1,2], Arthur Declercq[1,2], Emmy Terryn[1,2], Ralf Gabriels[1,2], Francis Impens[1,2], Kris Gevaert[1,2], Lennart Martens[1,2,3,4*], Robbin Bouwmeester[1,2]

1 VIB Center for Medical Biotechnology, VIB, Ghent, 9052, Belgium

2 Department of Biomolecular Medicine, Faculty of Medicine and Health Sciences, Ghent University, Ghent, 9052, Belgium

3 BioOrganic Mass Spectrometry Laboratory (LSMBO), IPHC UMR 7178, University of Strasbourg, CNRS, Strasbourg, 67000, France

4 Infrastructure Nationale de Protéomique ProFI – FR2048, Strasbourg, 67087, France

** corresponding author - email: lennart.martens@ugent.be*

Table of content

**Supplementary Methods**	1. Description of the custom loss function used to train the multi-output model.<br>2. Description of the methodology used to generate extracted ion mobilograms and detect peaks in the ion mobility distribution.
**Supplementary Table 1**	Overview of the public datasets used for further processing, along with the number of LC-IM-MS/MS runs and the sample species.
**Supplementary Figure 1**	Figure showing the processing workflow for the creation of the multiconformational precursor ion dataset
**Supplementary Table 2**	Overview of the hyperparameters of the final multi-output IM2Deep model.
**Supplementary Figure 2**	Figure showing the validation accuracies for models trained with difference weights from 0 to 10.
**Supplementary Figure 3**	Figure showing the distribution of conformer counts identified across multiple LC-IM-MS/MS runs, illustrating the impact of filtering on the final dataset.
**Supplementary Figure 4**	Figure showing the distribution of the number of conformers observed per peptidoform-charge state pair in the multiconformer dataset after filtering, for each charge state.
**Supplementary Figure 5**	Figure showing boxplots and countplot comparing the relative presence of all 20 amino acids between multiconformational and uniconformational peptides.
**Supplementary Figure 6**	Figure showing performance curves for the multi-output model, comparing performance between training the model from scratch and with the finetuning strategy.
**Supplementary Figure 7**	Figure showing histograms showing the distribution of absolute relative errors for CCS predictions of the multiconformational precursor test set.
**Supplementary Figure 8**	Figure showing scatter plots comparing predicted versus observed CCS values for the transfer-learned multi-output IM2Deep model, where performance on tryptic and non-tryptic precursor ions in the test set is plotted separately.
**Supplementary Figure 9**	Figure showing prediction accuracy comparison with the model from Meier *et al.* with both the original and multiconformational IM2Deep model.
**Supplementary Figure 10**	Figure showing bar plots visualizing the distribution of XIMs with one, two or more detected peaks across different intensity thresholds and technical replicates.
**Supplementary Figure 11**	Figure showing a scatter plot illustrating the relationship between the log-transformed ratio of IM peak intensities from two technical replicates.
**Supplementary Figure 12**	Figure showing an XIM from a precursor identified across three replicates, which exhibits two distinct peaks in the IM distribution, where the intensity order of the peaks differs across the three replicates.
**Supplementary Figure 13**	Figure showing mobilograms of selected precursors demonstrating remaining challenges in CCS prediction.

Supplementary methods

Custom loss function used to train the multi-output model

To ensure distinct predictions for each target, we implemented a custom loss function. Simple loss functions, such as the sum of mean absolute errors (MAEs) between each prediction-target pair, can lead to cases where both predictions converge to the same target. Our custom loss function was designed to address this issue by forcing the model to produce two distinct predictions corresponding to two separate targets. Firstly, both targets and model outputs are ordered by size to impose a consistent prediction-target pairing.

The mean absolute error is calculated between each ordered prediction and its corresponding ordered target:

$$L_1 = \frac{1}{N}\sum_{i=1}^{N}\left|\hat{y}_{(1)i} - y_{(1)i}\right| \tag{1}$$

$$L_2 = \frac{1}{N}\sum_{i=1}^{N}\left|\hat{y}_{(2)i} - y_{(2)i}\right| \tag{2}$$

where N is batch size and

$$\hat{y}_{(1)i} > \hat{y}_{(2)i} \tag{3}$$

$$y_{(1)i} > y_{(2)i} \tag{4}$$

Additionally, the difference between the targets and the predictions is computed:

$$D_y = \left|y_{(2)} - y_{(1)}\right| \tag{5}$$

$$D_{\hat{y}} = \left|\hat{y}_{(2)} - \hat{y}_{(1)}\right| \tag{6}$$

The mean absolute error of these differences is then calculated:

$$L_{diff} = \frac{1}{N}\sum_{i=1}^{N}\left|D_{\hat{y}i} - D_{yi}\right| \tag{7}$$

The total loss is then calculated as the sum of the two target-prediction MAEs and the MAE of the differences, multiplied by a weight to emphasize the importance of the difference term:

$$L_{total} = L_1 + L_2 + \alpha L_{diff} \tag{8}$$

This custom loss function ensures that each prediction corresponds closely to a distinct target. For the final models, this difference weight was set to six, as determined by achieving the highest accuracy on the validation set in a tuning experiment where the weight was set from zero up to ten (Supplementary Figure S2).

Extraction and visualization of ion mobilograms

To demonstrate the advantages of the multiconformational IM2Deep model and quantify the extent of multiconformationality in timsTOF data, we extracted ion mobilograms (XIMs) to visualize IM distributions for identified precursors from two public LC-IM-MS/MS datasets: one randomly selected run from PXD046507[1], to generate example ion mobilograms shown in Figure 7 and Supplementary Figure 13, and three replicate *E. coli* digest runs from PXD028735[2], used to perform the quantitative analysis on the multimodal behavior of XIMs. The raw timsTOF measurements were accessed from the .d folders using the AlphaTims (version 1.0.9) Python package.

For the PXD046507 data, we reused the MaxQuant evidence file associated with the original data. For the three replicates from PXD028735, the data was first searched using Sage (v0.14.3) with the following parameters: a 10 ppm precursor and fragment tolerance, oxidation of methionine, and acetylation of the protein N-terminus as variable modifications, and carbamidomethylation of cysteine as a fixed modification. Trypsin was used as the cleavage enzyme with a restriction for proline, allowing a maximum of one variable modification and two missed cleavages. The data was searched using the Swiss-Prot canonical *E. coli* proteome (UP000000625, 4,401 entries, downloaded June 2024), supplemented with common contaminant proteins.

The identified precursors were matched to the raw IM measurements based on their retention time and m/z values, with a retention time tolerance of 5 seconds and a precursor m/z tolerance of 10 ppm. These raw IM measurements were then converted to CCS values using the Mason-Schamp equation.

To detect peaks in the IM distributions of the precursors identified in the *E. coli* triplicate LC-IM-MS/MS runs, we applied the following peak detection algorithm: first, Gaussian filtering was applied to smooth the IM signal. Then, we used SciPy's find_peaks function (v1.11.4) to identify local maxima by comparing neighboring values in the smoothed intensity array. The peaks were filtered based on their properties, with different height thresholds applied: 10%, 25%, 50%, and 75% of the intensity of the highest peak. Additionally, peaks were required to have at least a 2% difference in CCS from the nearest higher peak.

Supplementary tables and figures

Supplementary Table 1: Overview of the public datasets used for further processing, including the number of LC-IM-MS/MS runs and the species from which the data were acquired.

PRIDE accession	Number of LC-IM-MS/MS runs	Species	Reference
**PXD019086**	459	*D. melanogaster*<br>*C. elegans*<br>*H. sapiens*<br>*E. coli*<br>*S. cerevisiae*	Meier *et al.* [3]
**PXD039469**	116	*H. sapiens*	Zila *et al.* [4]
**PXD042416**	84	*H. sapiens*	Will *et al.* [5]
**PXD035986**	72	*H. sapiens*	Romero-Gavilán *et al.* [6]
**PXD043511**	54	*H. sapiens*	Ries *et al.* [7]
**PXD036206**	48	*H. sapiens*	Pahmeier *et al.* [8]
**PXD037288**	47	*M. musculus*	Lamsal *et al.* [9]
**PXD036127**	42	*H. sapiens*	Ries *et al.* [10]
**PXD035987**	42	*H. sapiens*	Ries *et al.* [10]
**PXD038824**	41	*B. duttonii*<br>*O. moubata*	Filatov *et al.* [11]
**PXD042478**	34	*M. musculus*	Bradić *et al.* [12]
**PXD040890**	32	*R. norvegicus*	Puzio *et al.* [13]
**PXD037622**	28	*M. musculus*	de Jonckheere *et al.* [14]
**PXD049281**	16	*M. musculus*	Meulders *et al.* [15]
**PXD043166**	12	*E. granulosus*	Wang *et al.* [16]
**PXD042114**	12	*M. musculus*<br>*P. falciparum*	Mansour *et al.* [17]
**PXD035675**	12	*E. coli*	Zhu & Dai [18]
**PXD036970**	12	*H. sapiens*	Kovarik *et al.* [19]
**PXD036746**	12	*P. falciparum*	Scally *et al.* [20]
**PXD037945**	12	*M. musculus*	Huang *et al.* [21]
**PXD039646**	12	*P. falciparum*	Triglia *et al.* [22]
**PXD037089**	9	*C. savignyi*	Wang *et al.* [23]
**PXD040481**	8	*M. musculus*	Cao *et al.* [24]
**PXD043226**	6	*C. porcellus*	Feng *et al.* [25]
**PXD040521**	6	*S. scrofa domesticus*	Chen *et al.* [26]
**PXD038840**	6	*C. neoformans*	Li *et al.* [27]
**PXD048960**	6	*H. sapiens*	Xu *et al.* [28]
**PXD036191**	4	*H. sapiens*	Xu *et al.* [29]
**PXD036482**	2	*A. thaliana*	Klusch *et al.* [30]
**PXD043382**	2	*M. musculus*	-
**PXD038782**	366	*H. sapiens (immunopeptides)*	Hoenisch Gravel *et al.*[31]

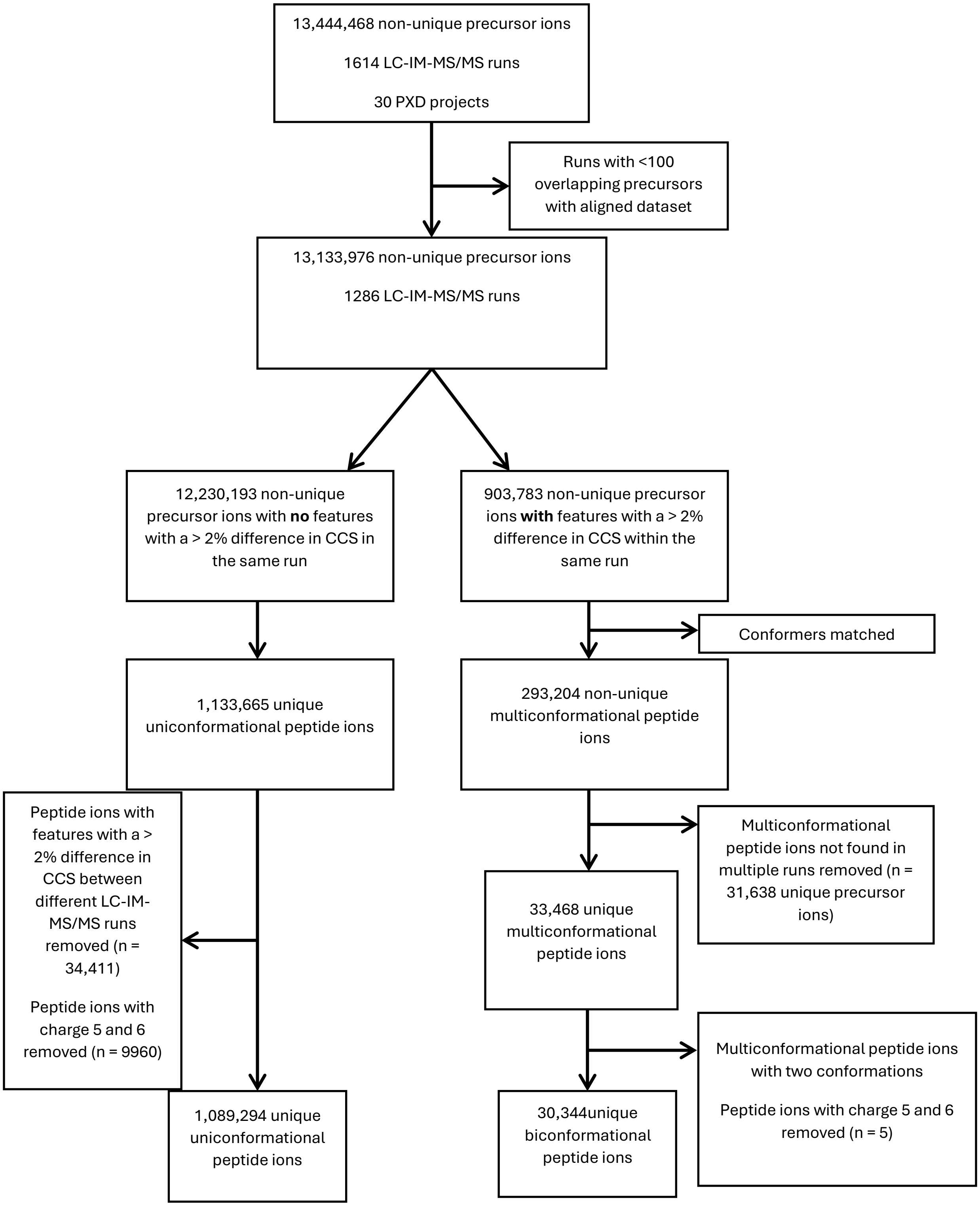

Supplementary Figure 1: Processing workflow for the creation of the multiconformational precursor ion dataset

Supplementary Table 2: Hyperparameters of the final multi-output IM2Deep model.

Hyperparameter	Value
**Batch size**	64
**Learning rate**	0.0001
**Activation function**	Tanh for one-hot encoding path, Leaky ReLU for other paths
**Leaky ReLU alpha**	0.1
**Maximum activation value**	20
**L1 lambda**	0.00001
**Max epochs**	500
**Kernel size**	4 for atom composition path, 2 for other paths
**Number of filters**	Atom composition path: 256<br>Diatom composition path: 128<br>One hot encoding path: 2
**Number of dense units**	Global features path: 16<br>Concatenated path: 128
**Difference weight**	6

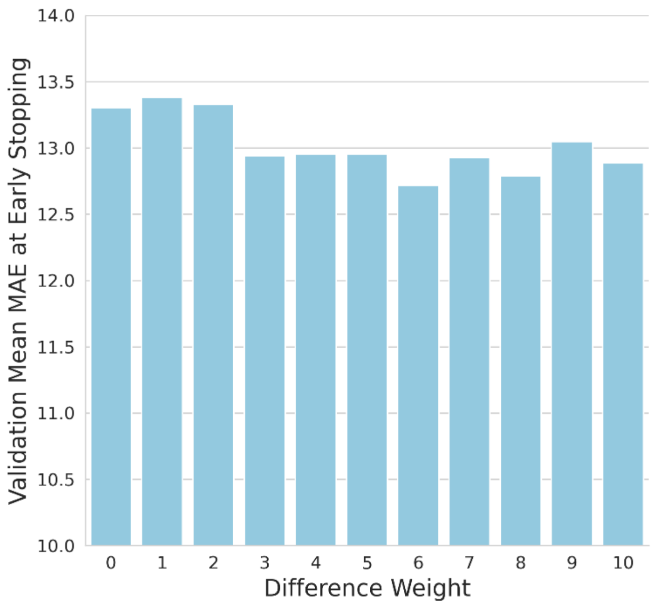

Supplementary Figure 2: Validation mean MAE for models trained with difference weights from 0 to 10.

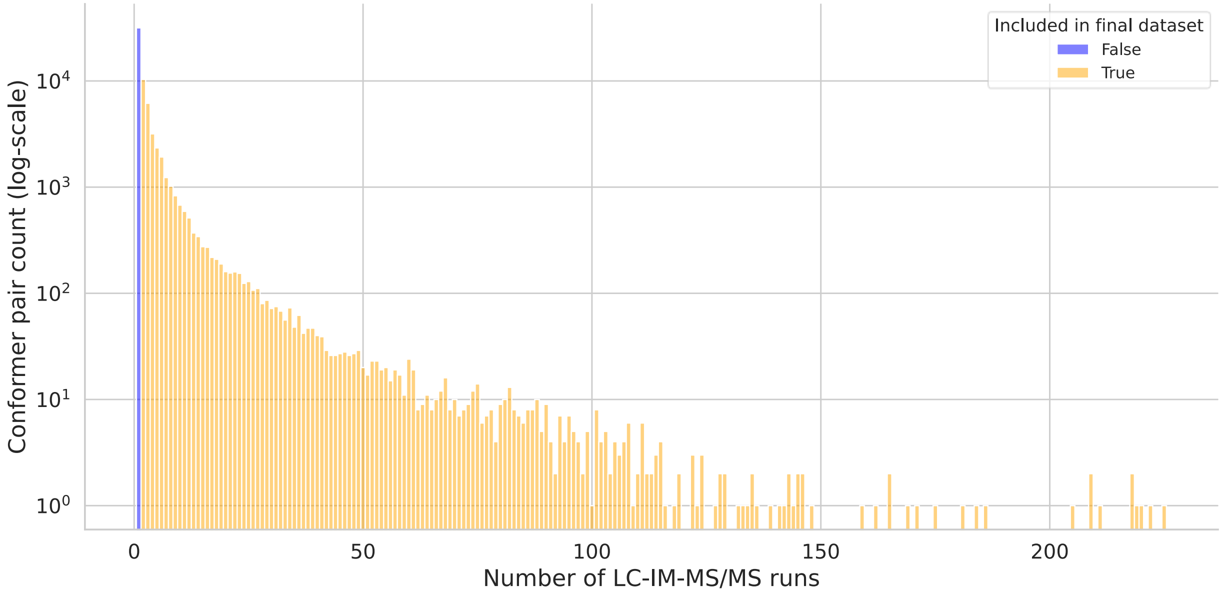

Supplementary Figure 3: Distribution of conformer counts identified across multiple LC-IM-MS/MS runs, illustrating the impact of filtering on the final dataset. Horizontal axis represents the number of runs in which a conformer pairing was identified, while the vertical axis (log-scale) shows the total number of conformer pairs. Yellow bars represent conformers that were included in the final dataset after filtering.

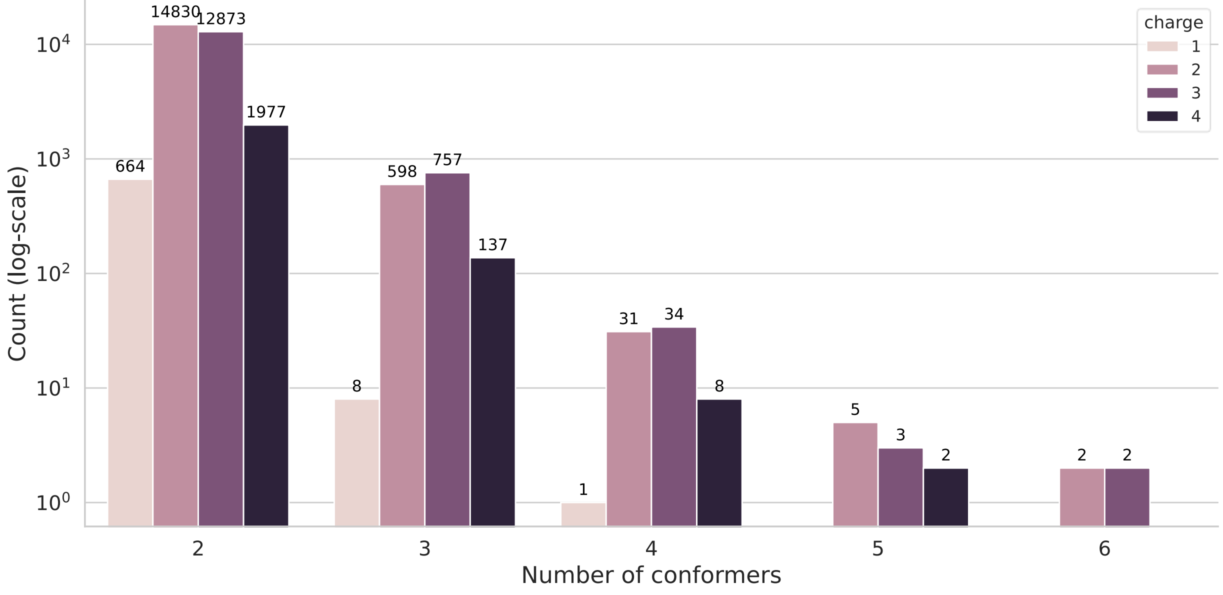

Supplementary Figure 4: Distribution of the number of conformers observed per peptidoform-charge state pair in the multiconformer dataset after filtering, for each charge state. Horizontal-axis represents the number of conformers identified for each precursor, and the vertical-axis (log-scale) shows the count of peptidoform-charge state pairs.

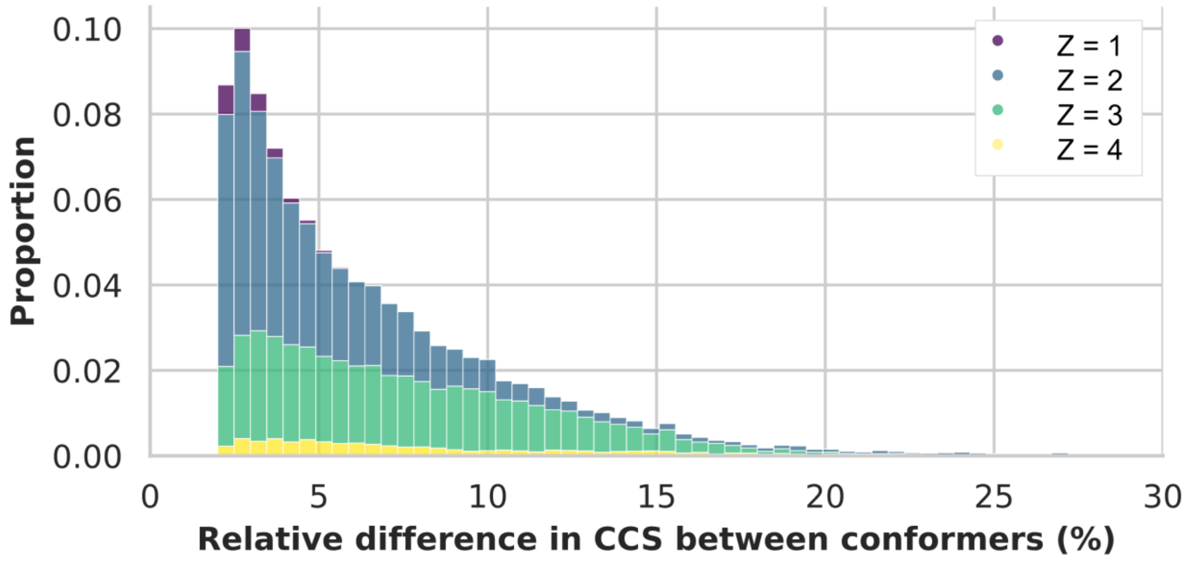

Supplementary Figure 5: Stacked histogram showing the distribution of relative differences in CCS between conformers for peptide ions with different charge states.

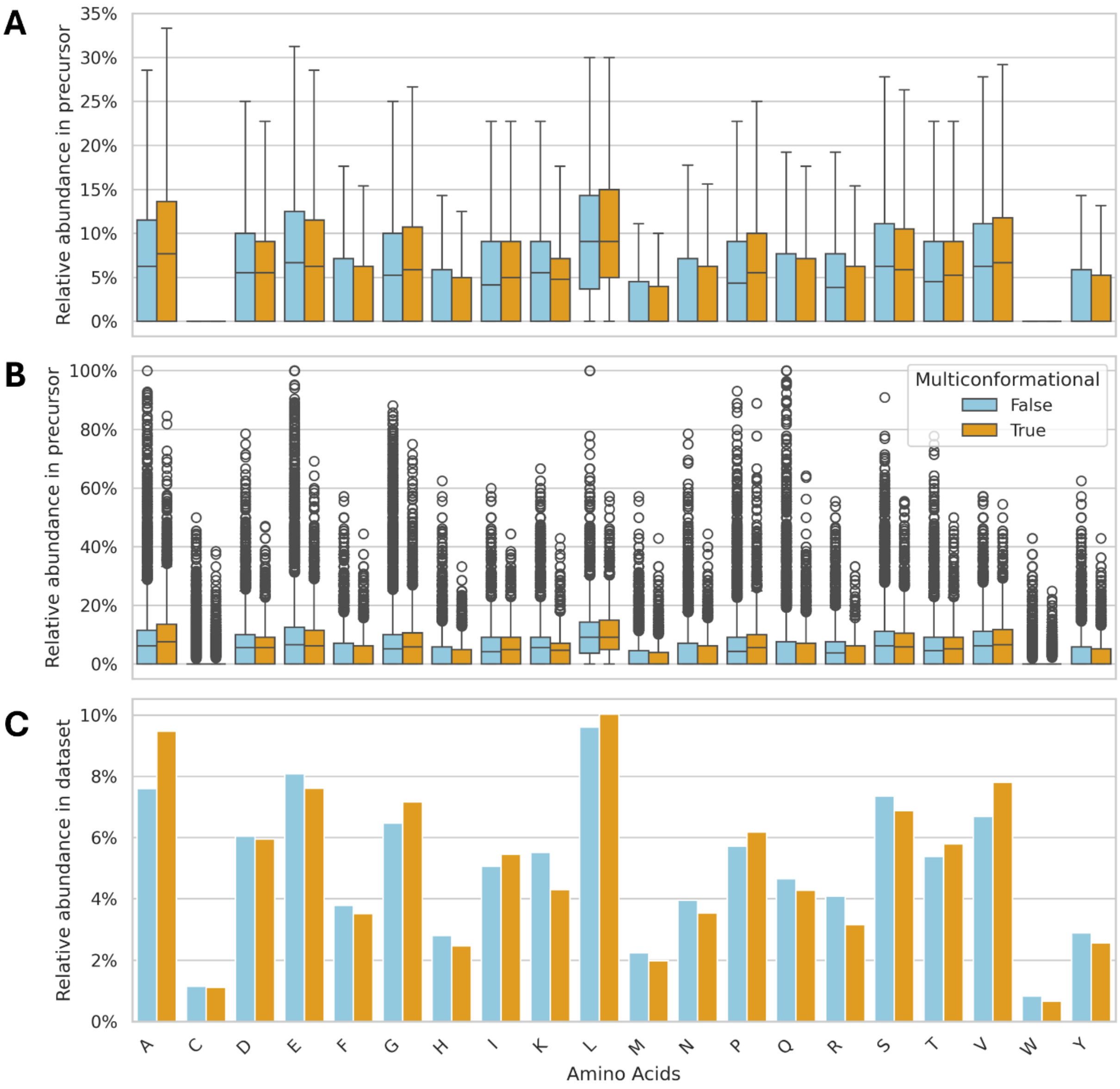

Supplementary Figure 6: A–B) Boxplots showing the relative abundance of each of the 20 amino acids within individual precursor sequences, compared between multiconformational (orange) and uniconformational (blue) peptides. Panel A displays boxplots with outliers hidden; panel B includes outliers. The relative abundance within a precursor is calculated by dividing the count of each amino acid by the total length of the precursor sequence. C) Barplot comparing the overall relative abundance of each of the 20 amino acids across entire datasets of multiconformational (orange) and uniconformational (blue) peptides. The relative abundance per dataset is computed by dividing the total count of each amino acid (summed across all sequences) by the cumulative length of all precursor sequences in that dataset.

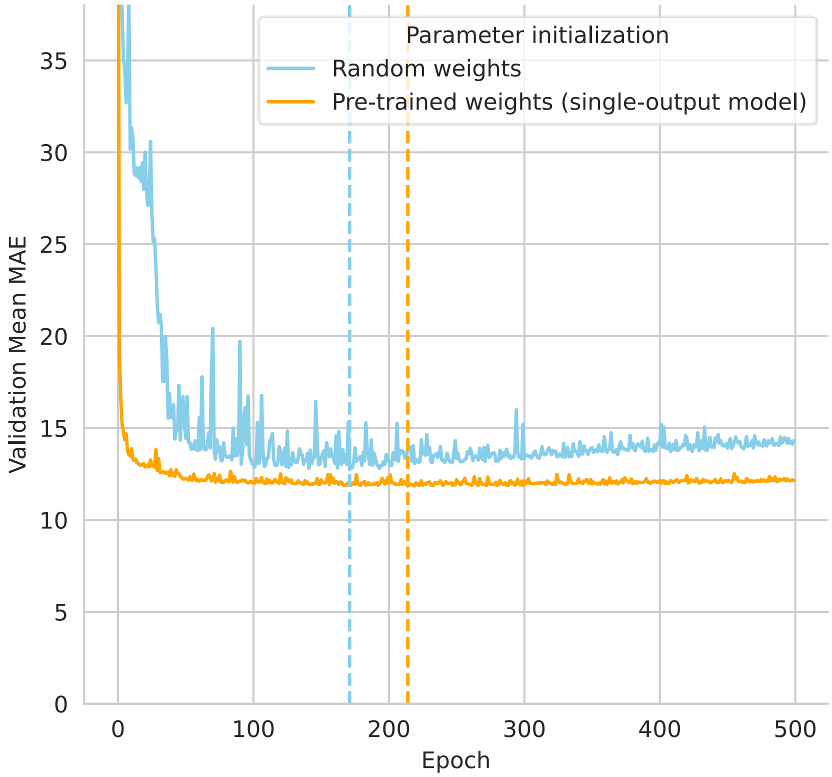

Supplementary Figure 7: Performance curves over 500 training epochs for the multi-output IM2Deep model, comparing performance with different parameter initialization strategies. The model initialized with pre-trained weights from a single-output IM2Deep model (orange) shows consistently lower validation MAE compared to the model initialized with random weights (blue). Dotted lines represent the points at which the highest validation accuracy is achieved, i.e., where early stopping occurs.

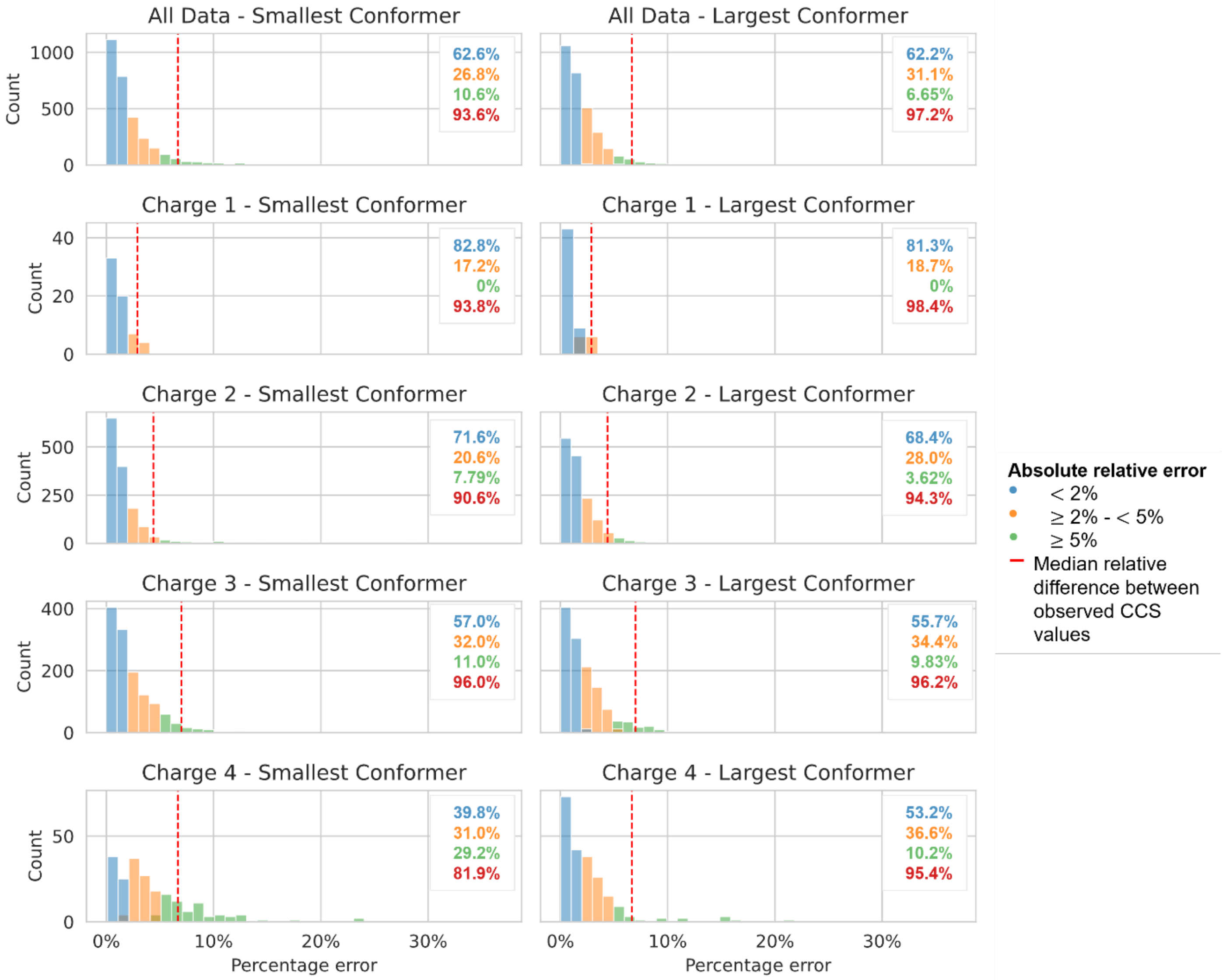

Supplementary Figure 8: Distribution of absolute relative errors for CCS predictions of the multiconformational peptide test set. The upper panel shows the distribution of absolute relative errors across the full multiconformational peptide test set, separately for the smaller and larger conformers. Lower panels show the same distributions stratified by precursor charge state. Vertical red dotted lines represent the median relative difference between observed CCS values within each subgroup. The percentages in each subplot denote the proportion of predictions below 2% (blue), between 2 and 5% (orange) and over 5% (green), and the proportion of predictions with errors below the median relative difference between CCS values (red).

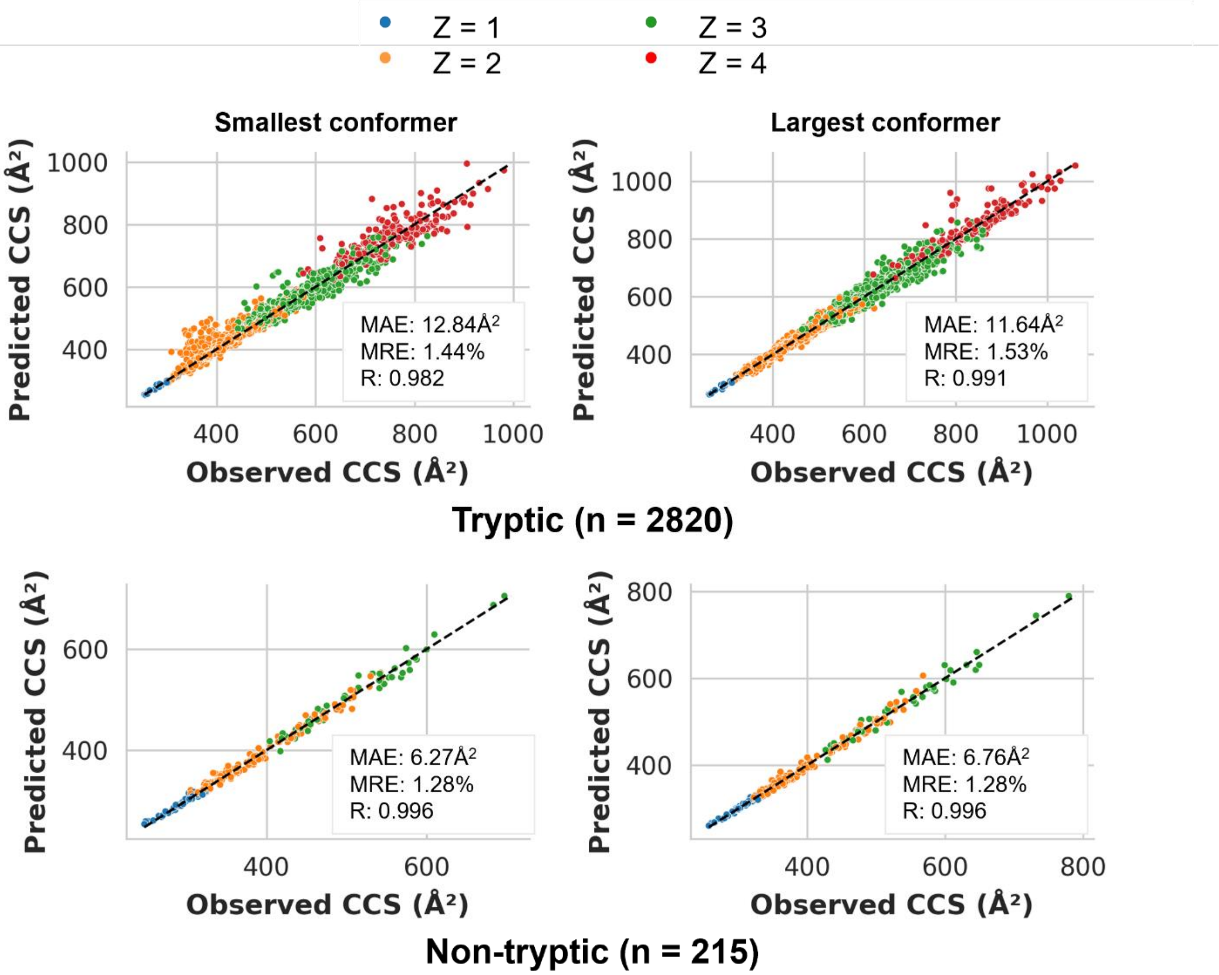

Supplementary Figure 9: Scatter plots comparing predicted versus observed CCS values for the transfer-learned multi-output IM2Deep model, where performance on tryptic (upper) and non-tryptic (lower) precursor ions in the test set are plotted separately.

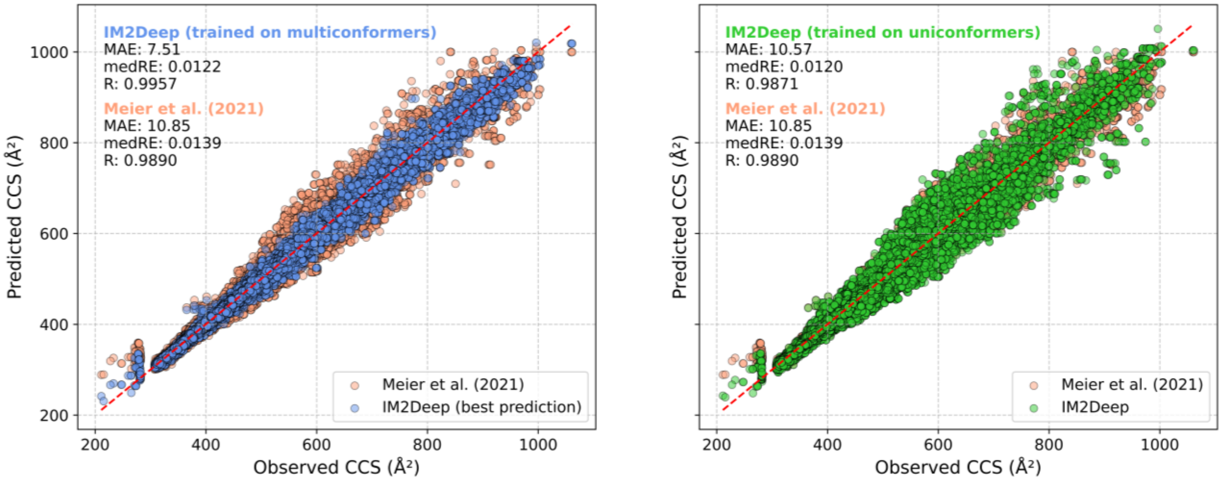

Supplementary Figure 10: Performance comparison with the model from Meier *et al*. Left panel shows comparison between the multi-output IM2Deep model trained on multiconformers, where the best prediction is selected. The right panel shows comparison with the single-output IM2Deep model trained on uniconformers.

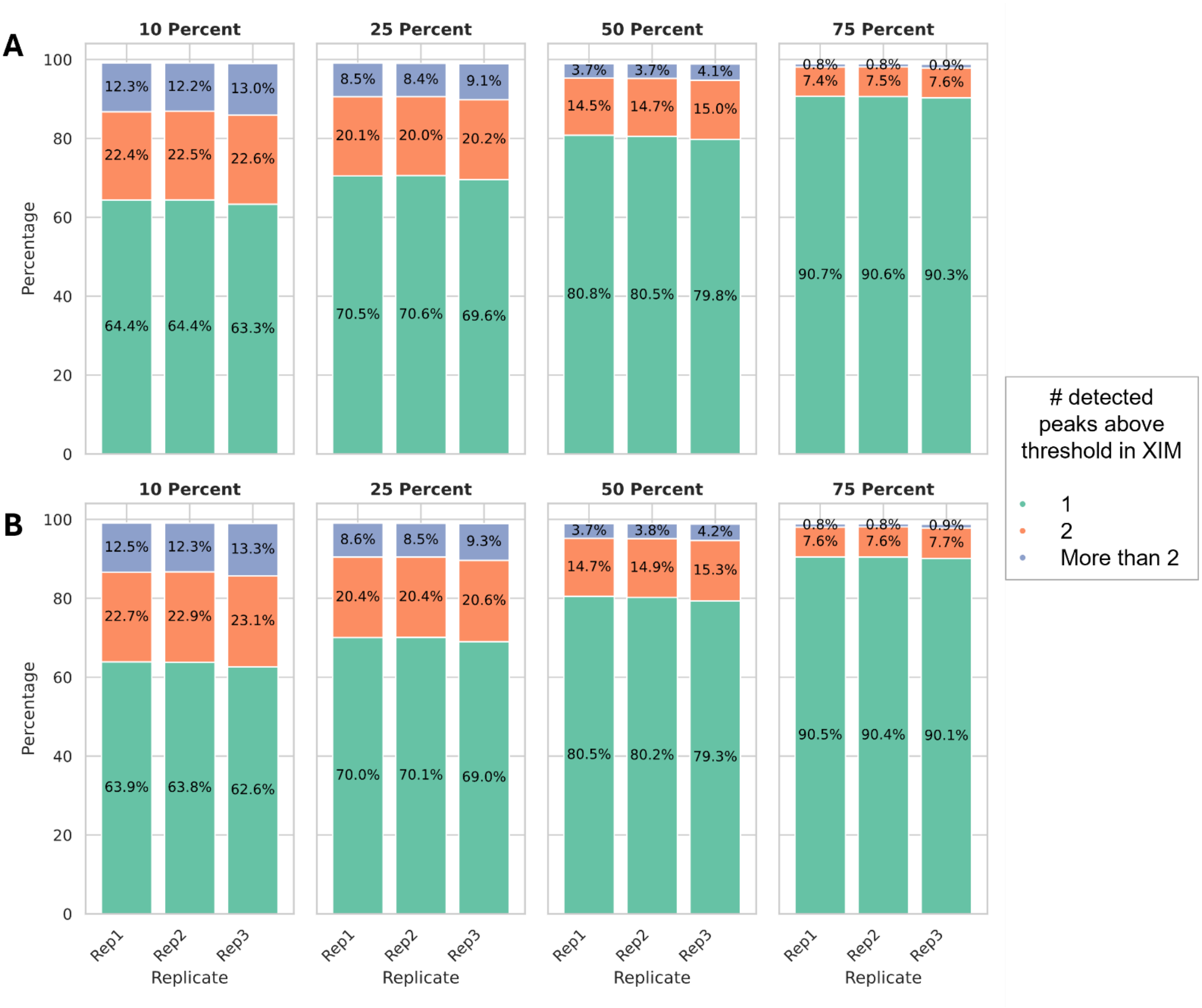

Supplementary Figure 11: Bar plots showing the distribution of extracted ion mobilograms (XIMs) with one (green), two (orange), or more than two (blue) detected peaks across different intensity thresholds and technical replicates. (A) All precursor XIMs are included. (B) Only XIMs from precursors identified in all three technical replicates are considered. Each panel (10%, 25%, 50%, 75%) represents a different peak intensity threshold, meaning that any secondary peaks must be at least that percentage of the intensity of the main peak to be counted.

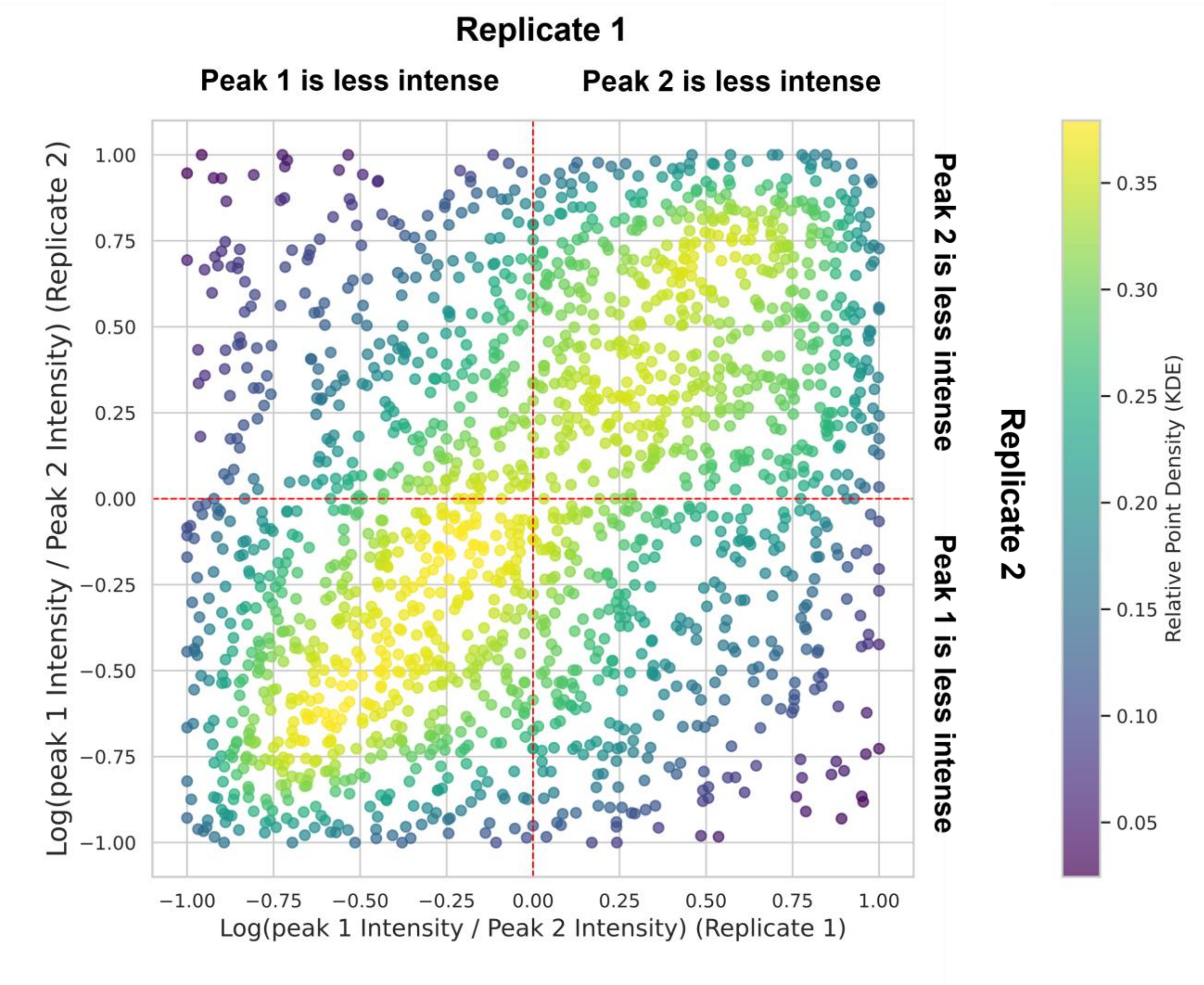

Supplementary Figure 12: Scatter plot illustrating the relationship between the log-transformed ratio of IM peak intensities from two technical replicates. In this plot, peak 1 corresponds to the peak with the lower CCS value, and peak 2 corresponds to the peak with the higher CCS value. Each point represents a precursor's ion mobilogram extracted in both replicates, and its color reflects the point density in that region, with denser areas shown in yellow and sparser areas in purple. The red lines indicate the quadrants of the plot. Points in the upper right and lower left quadrants represent precursors where the IM peak intensities in the extracted ion mobilogram maintain the same order across both replicates. In contrast, points in the upper left and lower right quadrants indicate precursors where the order of IM peak intensities has reversed between replicates.

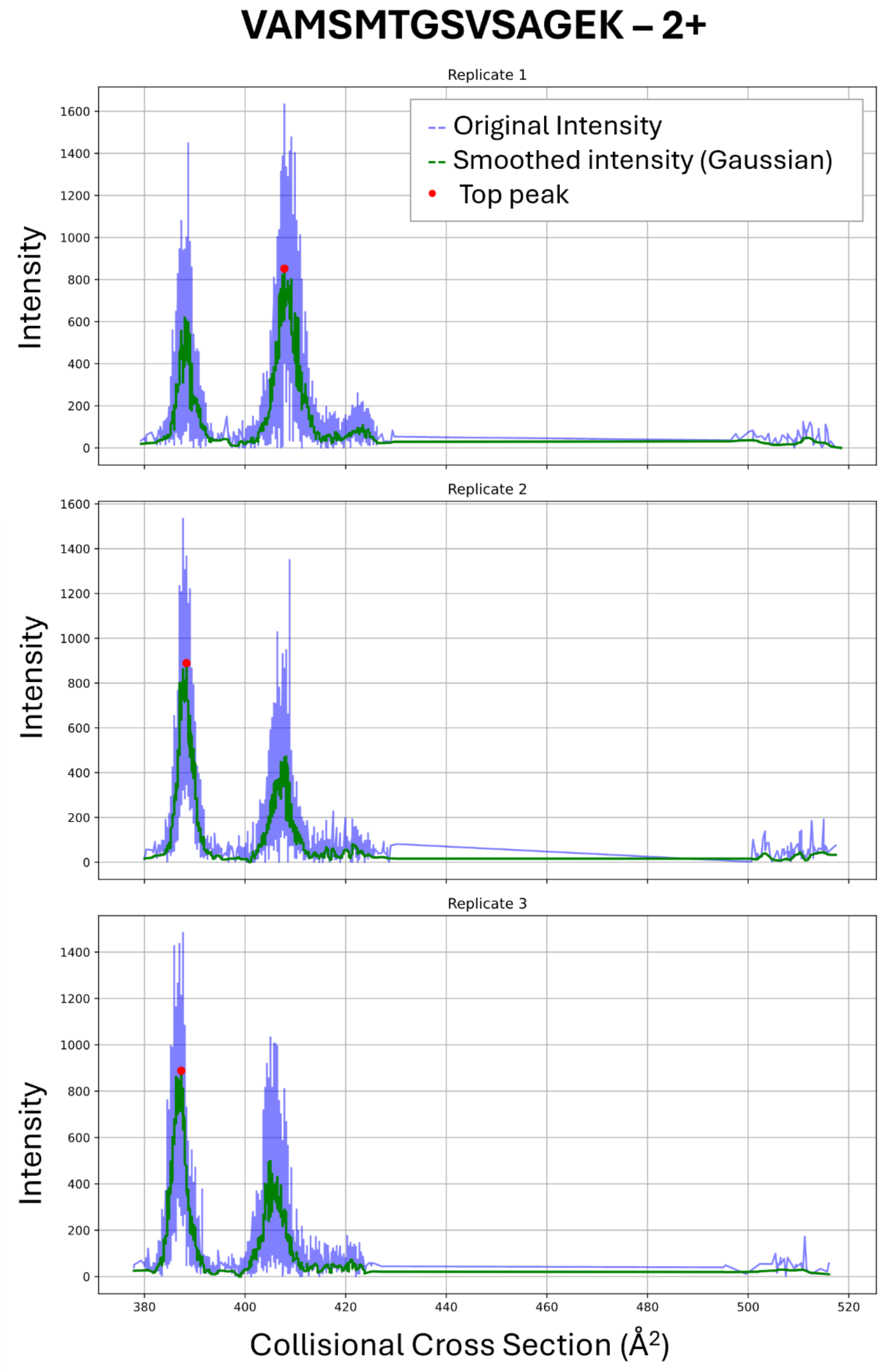

Supplementary Figure 13: Extracted ion mobilograms from a precursor identified in the PXD028735 *E. coli* data (UniProt ID of inferred protein: P25553) across three replicates. The precursor exhibits two distinct peaks in the IM distribution, where the intensity order of the peaks differs across the three replicates.

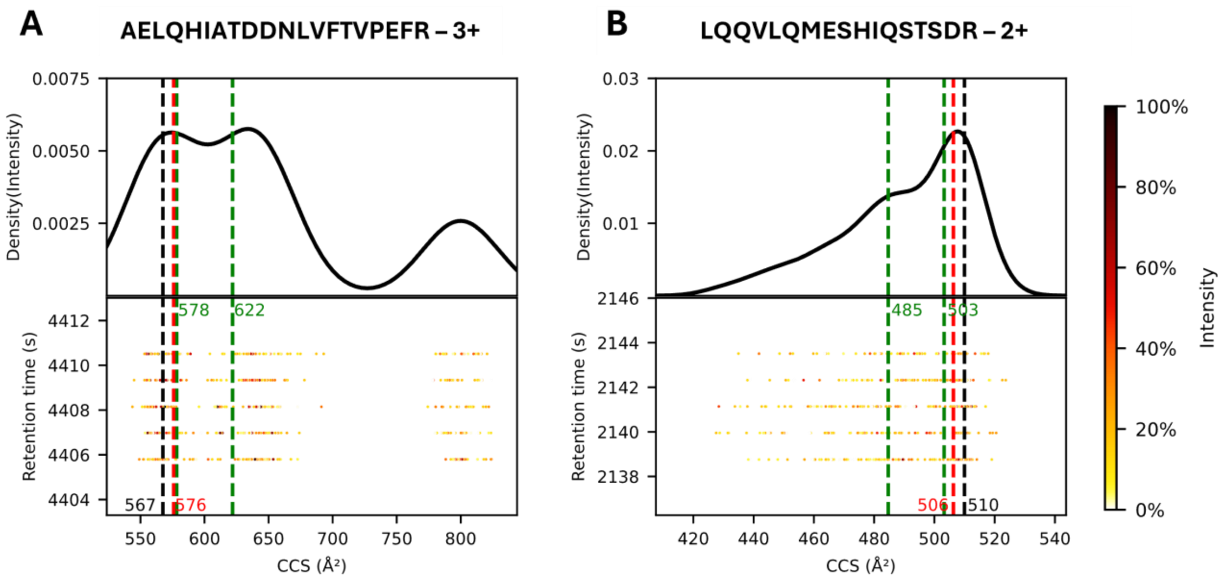

Supplementary Figure 14: Mobilograms of selected precursor ions from PXD046507 demonstrating remaining challenges in CCS prediction. A) Precursor ion (UniProt ID: P12111) clearly exhibiting three peaks in the ion mobility distribution. B) This peptide ion (UniProt ID: Q14974) exhibits an ion mobilogram with unclear distinction between conformers, especially in the shoulder of the main conformation. CCS value reported by MaxQuant is indicated by a black dashed line. Classical IM2Deep prediction (single-output) indicated by red dashed line. CCS predictions made by multiconformational model indicated by green dashed lines.

Supplementary references

(1) Hu, Z.; Wu, Z.; Liu, W.; Ning, Y.; Liu, J.; Ding, W.; Fan, J.; Cai, S.; Li, Q.; Li, W.; Yang, X.; Dou, Y.; Wang, W.; Peng, W.; Lu, F.; Zhuang, X.; Qin, T.; Kang, X.; Feng, C.; Xu, Z.; Lv, Q.; Wang, Q.; Wang, C.; Wang, X.; Wang, Z.; Wang, J.; Jiang, J.; Wang, B.; Mills, G. B.; Ma, D.; Gao, Q.; Li, K.; Chen, G.; Chen, X.; Sun, C. Proteogenomic Insights into Early-Onset Endometrioid Endometrial Carcinoma: Predictors for Fertility-Sparing Therapy Response. *Nat Genet* **2024**, *56* (4), 637–651. https://doi.org/10.1038/s41588-024-01703-z.

(2) Van Puyvelde, B.; Daled, S.; Willems, S.; Gabriels, R.; Gonzalez de Peredo, A.; Chaoui, K.; Mouton-Barbosa, E.; Bouyssié, D.; Boonen, K.; Hughes, C. J.; Gethings, L. A.; Perez-Riverol, Y.; Bloomfield, N.; Tate, S.; Schiltz, O.; Martens, L.; Deforce, D.; Dhaenens, M. A Comprehensive LFQ Benchmark Dataset on Modern Day Acquisition Strategies in Proteomics. *Sci Data* **2022**, *9* (1). https://doi.org/10.1038/s41597-022-01216-6.

(3) Meier, F.; Köhler, N. D.; Brunner, A. D.; Wanka, J. M. H.; Voytik, E.; Strauss, M. T.; Theis, F. J.; Mann, M. Deep Learning the Collisional Cross Sections of the Peptide Universe from a Million Experimental Values. *Nat Commun* **2021**, *12* (1). https://doi.org/10.1038/s41467-021-21352-8.

(4) Zila, N.; Eichhoff, O. M.; Steiner, I.; Mohr, T.; Bileck, A.; Cheng, P. F.; Leitner, A.; Gillet, L.; Sajic, T.; Goetze, S.; Friedrich, B.; Bortel, P.; Strobl, J.; Reitermaier, R.; Hogan, S. A.; Martínez Gómez, J. M.; Staeger, R.; Tuchmann, F.; Peters, S.; Stary, G.; Kuttke, M.; Elbe-Bürger, A.; Hoeller, C.; Kunstfeld, R.; Weninger, W.; Wollscheid, B.; Dummer, R.; French, L. E.; Gerner, C.; Aebersold, R.; Levesque, M. P.; Paulitschke, V. Proteomic Profiling of Advanced Melanoma Patients to Predict Therapeutic Response to Anti-PD-1 Therapy. *Clinical Cancer Research* **2024**, *30* (1), 159–175. https://doi.org/10.1158/1078-0432.CCR-23-0562.

(5) Will, A.; Oliinyk, D.; Bleiholder, C.; Meier, F. Peptide Collision Cross Sections of 22 Post-Translational Modifications. *Anal Bioanal Chem* **2023**, *415* (27), 6633–6645. https://doi.org/10.1007/s00216-023-04957-4.

(6) Romero-Gavilán, F.; Cerqueira, A.; García-Arnáez, I.; Azkargorta, M.; Elortza, F.; Gurruchaga, M.; Goñi, I.; Suay, J. Proteomic Evaluation of Human Osteoblast Responses to Titanium Implants over Time. *J Biomed Mater Res A* **2023**, *111* (1), 45–59. https://doi.org/10.1002/jbm.a.37444.

(7) Ries, A.; Slany, A.; Pirker, C.; Mader, J. C.; Mejri, D.; Mohr, T.; Schelch, K.; Flehberger, D.; Maach, N.; Hashim, M.; Hoda, M. A.; Dome, B.; Krupitza, G.; Berger, W.; Gerner, C.; Holzmann, K.; Grusch, M. Primary and HTERT-Transduced Mesothelioma-Associated Fibroblasts but Not Primary or HTERT-Transduced Mesothelial Cells Stimulate Growth of Human Mesothelioma Cells. *Cells* **2023**, *12* (15). https://doi.org/10.3390/cells12152006.

(8) Pahmeier, F.; Lavacca, T.-M.; Goellner, S.; Neufeldt, C. J.; Prasad, V.; Cerikan, B.; Rajasekharan, S.; Mizzon, G.; Haselmann, U.; Funaya, C.; Scaturro, P.; Cortese, M.; Bartenschlager, R. Identification of Host Dependency Factors Involved in SARS-CoV-2 Replication Organelle Formation through Proteomics and Ultrastructural Analysis. *J Virol* **2023**, *97* (11). https://doi.org/10.1128/jvi.00878-23.

(9) Lamsal, A.; Andersen, S. B.; Johansson, I.; Vietri, M.; Bokil, A. A.; Kurganovs, N. J.; Rylander, F.; Bjørkøy, G.; Pettersen, K.; Giambelluca, M. S. Opposite and Dynamic Regulation of the Interferon Response in Metastatic and Non-Metastatic Breast Cancer. *Cell Communication and Signaling* **2023**, *21* (1). https://doi.org/10.1186/s12964-023-01062-y.

(10) Ries, A.; Flehberger, D.; Slany, A.; Pirker, C.; Mader, J. C.; Mohr, T.; Schelch, K.; Sinn, K.; Mosleh, B.; Hoda, M. A.; Dome, B.; Dolznig, H.; Krupitza, G.; Müllauer, L.; Gerner, C.; Berger, W.; Grusch, M. Mesothelioma-Associated Fibroblasts Enhance Proliferation and Migration of Pleural Mesothelioma Cells via c-Met/PI3K and WNT Signaling but Do Not Protect against Cisplatin. *Journal of Experimental and Clinical Cancer Research* **2023**, *42* (1). https://doi.org/10.1186/s13046-022-02582-0.

(11) Filatov, S.; Dyčka, F.; Sterba, J.; Rego, R. O. M. A Simple Non-Invasive Method to Collect Soft Tick Saliva Reveals Differences in Ornithodoros Moubata Saliva Composition between Ticks Infected and Uninfected with Borrelia Duttonii Spirochetes. *Front Cell Infect Microbiol* **2023**, *13*. https://doi.org/10.3389/fcimb.2023.1112952.

(12) Bradić, I.; Liesinger, L.; Kuentzel, K. B.; Vujić, N.; Trauner, M.; Birner-Gruenberger, R.; Kratky, D. Metabolic Changes and Propensity for Inflammation, Fibrosis, and Cancer in Livers of Mice Lacking Lysosomal Acid Lipase. *J Lipid Res* **2023**, *64* (9). https://doi.org/10.1016/j.jlr.2023.100427.

(13) Puzio, M.; Moreton, N.; Sullivan, M.; Scaife, C.; Glennon, J. C.; O'Connor, J. J. An Electrophysiological and Proteomic Analysis of the Effects of the Superoxide Dismutase Mimetic, MnTMPyP, on Synaptic Signalling Post-Ischemia in Isolated Rat Hippocampal Slices. *Antioxidants* **2023**, *12* (4). https://doi.org/10.3390/antiox12040792.

(14) de Jonckheere, B.; Kollotzek, F.; Münzer, P.; Göb, V.; Fischer, M.; Mott, K.; Coman, C.; Troppmair, N. N.; Manke, M. C.; Zdanyte, M.; Harm, T.; Sigle, M.; Kopczynski, D.; Bileck, A.; Gerner, C.; Hoffmann, N.; Heinzmann, D.; Assinger, A.; Gawaz, M.; Stegner, D.; Schulze, H.; Borst, O.; Ahrends, R. Critical Shifts in Lipid Metabolism Promote Megakaryocyte Differentiation and Proplatelet Formation. *Nature Cardiovascular Research* **2023**, *2* (9), 835–852. https://doi.org/10.1038/s44161-023-00325-8.

(15) Meulders, B.; Marei, W. F. A.; Xhonneux, I.; Loier, L.; Smits, A.; Leroy, J. L. M. R. Preconception Diet Interventions in Obese Outbred Mice and the Impact on Female Offspring Metabolic Health and Oocyte Quality. *Int J Mol Sci* **2024**, *25* (4). https://doi.org/10.3390/ijms25042236.

(16) Wang, Z.; Jia, X.; Ma, J.; Zhang, Y.; Sun, Y.; Bo, X. Global Profiling of the Proteome, Phosphoproteome, and N-Glycoproteome of Protoscoleces and Adult Worms of Echinococcus Granulosus. *Front Vet Sci* **2023**, *10*. https://doi.org/10.3389/fvets.2023.1275486.

(17) Mansour, H.; Cabezas-Cruz, A.; Peucelle, V.; Farce, A.; Salomé-Desnoulez, S.; Metatla, I.; Guerrera, I. C.; Hollin, T.; Khalife, J. Characterization of GEXP15 as a Potential Regulator of Protein Phosphatase 1 in Plasmodium Falciparum. *Int J Mol Sci* **2023**, *24* (16). https://doi.org/10.3390/ijms241612647.

(18) Zhu, M.; Dai, X. Stringent Response Ensures the Timely Adaptation of Bacterial Growth to Nutrient Downshift. *Nat Commun* **2023**, *14* (1). https://doi.org/10.1038/s41467-023-36254-0.

(19) Kovarik, J. J.; Bileck, A.; Hagn, G.; Meier-Menches, S. M.; Frey, T.; Kaempf, A.; Hollenstein, M.; Shoumariyeh, T.; Skos, L.; Reiter, B.; Gerner, M. C.; Spannbauer, A.; Hasimbegovic, E.; Schmidl, D.; Garhöfer, G.; Gyöngyösi, M.; Schmetterer, K. G.; Gerner, C. A Multi-Omics Based Anti-Inflammatory Immune Signature Characterizes Long COVID-19 Syndrome. *iScience* **2023**, *26* (1). https://doi.org/10.1016/j.isci.2022.105717.

(20) Scally, S. W.; Triglia, T.; Evelyn, C.; Seager, B. A.; Pasternak, M.; Lim, P. S.; Healer, J.; Geoghegan, N. D.; Adair, A.; Tham, W. H.; Dagley, L. F.; Rogers, K. L.; Cowman, A. F. PCRCR Complex Is Essential for Invasion of Human Erythrocytes by Plasmodium Falciparum. *Nat Microbiol* **2022**, *7* (12), 2039–2053. https://doi.org/10.1038/s41564-022-01261-2.

(21) Huang, Q.; Sun, Y.; Sun, J.; Peng, L.; Shang, H.; Wei, D.; Li, C.; Hu, Z.; Peng, H. Proteomic Characterization of Peritoneal Extracellular Vesicles in a Mouse Model of Peritoneal Fibrosis. *J Proteome Res* **2023**, *22* (3), 908–918. https://doi.org/10.1021/acs.jproteome.2c00713.

(22) Triglia, T.; Scally, S. W.; Seager, B. A.; Pasternak, M.; Dagley, L. F.; Cowman, A. F. Plasmepsin X Activates the PCRCR Complex of Plasmodium Falciparum by Processing PfRh5 for Erythrocyte Invasion. *Nat Commun* **2023**, *14* (1). https://doi.org/10.1038/s41467-023-37890-2.

(23) Wang, Z.; Ouyang, X.; Tan, Z.; Yang, L.; Dong, B. Quantitative Phosphoproteomics Reveals the Requirement of DYRK1-Mediated Phosphorylation of Ion Transport- and Cell Junction-Related Proteins for Notochord Lumenogenesis in Ascidian. *Cells* **2023**, *12* (6). https://doi.org/10.3390/cells12060921.

(24) Cao, H.; Cai, Q.; Guo, W.; Su, Q.; Qin, H.; Wang, T.; Xian, Y.; Zeng, L.; Cai, M.; Guan, H.; Chen, S.; Liang, H.; Xu, F. Malonylation of Acetyl-CoA Carboxylase 1 Promotes Hepatic Steatosis and Is Attenuated by Ketogenic Diet in NAFLD. *Cell Rep* **2023**, *42* (4). https://doi.org/10.1016/j.celrep.2023.112319.

(25) Feng, J.; Zhang, X.; Li, R.; Zhao, P.; Han, X.; Wu, Q.; Tian, Q.; Tang, G.; Song, J.; Bi, H. Widespread Involvement of Acetylation in the Retinal Metabolism of Form-Deprivation Myopia in Guinea Pigs. *ACS Omega* **2023**, *8* (26), 23825–23839. https://doi.org/10.1021/acsomega.3c02219.

(26) Chen, X.; Wang, S.; Wu, M.; Zhao, Y. Role of Succinylation in Pseudorabies Virus Infection. *J Virol* **2023**, *97* (4). https://doi.org/10.1128/jvi.01790-22.

(27) Li, C.; Meng, Y.; Li, H.; Du, W.; Gao, X.; Suo, C.; Gao, Y.; Ni, Y.; Sun, T.; Yang, S.; Lan, T.; Xin, M.; Ding, C. Immunization with a Heat-Killed Prm1 Deletion Strain Protects the Host from Cryptococcus Neoformans Infection. *Emerg Microbes Infect* **2023**, *12* (2). https://doi.org/10.1080/22221751.2023.2244087.

(28) Xu, L.; Li, W.; Liu, D.; Cao, J.; Ge, J.; Liu, X.; Wang, Y.; Teng, Y.; Liu, P.; Guo, X.; He, C.; Liu, M.; Tian, L. ANXA3-Rich Exosomes Derived from Tumor-Associated Macrophages Regulate

Ferroptosis and Lymphatic Metastasis of Laryngeal Squamous Cell Carcinoma. *Cancer Immunol Res* **2024**, *12* (5), 614–630. https://doi.org/10.1158/2326-6066.CIR-23-0595.

(29) Xu, T.; Ma, Q.; Li, Y.; Yu, Q.; Pan, P.; Zheng, Y.; Li, Z.; Xiong, X.; Hou, T.; Yu, B.; Liu, H.; Sun, Y. A Small Molecule Inhibitor of the UBE2F-CRL5 Axis Induces Apoptosis and Radiosensitization in Lung Cancer. *Signal Transduct Target Ther* **2022**, *7* (1). https://doi.org/10.1038/s41392-022-01182-w.

(30) Klusch, N.; Dreimann, M.; Senkler, J.; Rugen, N.; Kühlbrandt, W.; Braun, H. P. Cryo-EM Structure of the Respiratory I + III2 Supercomplex from Arabidopsis Thaliana at 2 Å Resolution. *Nat Plants* **2023**, *9* (1), 142–156. https://doi.org/10.1038/s41477-022-01308-6.

(31) Hoenisch Gravel, N.; Nelde, A.; Bauer, J.; Mühlenbruch, L.; Schroeder, S. M.; Neidert, M. C.; Scheid, J.; Lemke, S.; Dubbelaar, M. L.; Wacker, M.; Dengler, A.; Klein, R.; Mauz, P. S.; Löwenheim, H.; Hauri-Hohl, M.; Martin, R.; Hennenlotter, J.; Stenzl, A.; Heitmann, J. S.; Salih, H. R.; Rammensee, H. G.; Walz, J. S. TOFIMS Mass Spectrometry-Based Immunopeptidomics Refines Tumor Antigen Identification. *Nat Commun* **2023**, *14* (1). https://doi.org/10.1038/s41467-023-42692-7.
